# Development of Oxadiazole-Based ODZ10117 as a Small-Molecule Inhibitor of STAT3 for Targeted Cancer Therapy

**DOI:** 10.3390/jcm8111847

**Published:** 2019-11-02

**Authors:** Byung-Hak Kim, Haeri Lee, Yeonghun Song, Joon-Suk Park, Changdev G. Gadhe, Jiwon Choi, Chung-Gi Lee, Ae Nim Pae, Sanghee Kim, Sang-Kyu Ye

**Affiliations:** 1 Department of Pharmacology and Biomedical Sciences, Seoul National University College of Medicine, Seoul 03080, Korea; protein0826@snu.ac.kr (B.-H.K.); hrlee519@snu.ac.kr (H.L.); c_jiwon@hotmail.com (J.C.); 2 Biomedical Science Project (BK21PLUS), Seoul National University College of Medicine, Seoul 03080, Korea; 3 CYTUS H&B Corporation, Cheongju 28160, Korea; cglee2020@gmail.com; 4 College of Pharmacy, Seoul National University, Seoul 08826, Korea; song2@snu.ac.kr (Y.S.); pennkim@snu.ac.kr (S.K.); 5 Laboratory Animal Center, Daegu-Gyeongbuk Medical Innovation Foundation, Daegu 41061, Korea; 6 Convergence Research Center for Diagnosis, Treatment and Care System of Dementia, Korea Institute of Science and Technology, Seoul 02792, Korea; gadhe.changdev@gmail.com (C.G.G.); anpae@kist.re.kr (A.N.P.); 7 Division of Bio-Medical Science &Technology, KIST School, Korea University of Science and Technology, Seoul 02792, Korea; 8 Ischemic/Hypoxic Disease Institute, Seoul National University College of Medicine, Seoul 03080, Korea; 9 Neuro-Immune Information Storage Network Research Center, Seoul National University College of Medicine, Seoul 03080, Korea

**Keywords:** 3-(2,4-Dichloro-phenoxymethyl)-5-trichloromethyl-[1,2,4]oxadiazole (ODZ10117), STAT3, SH2 domain, targeted therapy, structure-based computational database screening, cell-based high-throughput screening

## Abstract

Persistently activated STAT3 is a promising target for a new class of anticancer drug development and cancer therapy, as it is associated with tumor initiation, progression, malignancy, drug resistance, cancer stem cell properties, and recurrence. Here, we discovered 3-(2,4-dichloro-phenoxymethyl)-5-trichloromethyl-[1,2,4]oxadiazole (ODZ10117) as a small-molecule inhibitor of STAT3 to be used in STAT3-targeted cancer therapy. ODZ10117 targeted the SH2 domain of STAT3 regardless of other STAT family proteins and upstream regulators of STAT3, leading to inhibition of the tyrosine phosphorylation, dimerization, nuclear translocation, and transcriptional activity of STAT3. The inhibitory effect of ODZ10117 on STAT3 was stronger than the known STAT3 inhibitors such as S3I-201, STA-21, and nifuroxazide. ODZ10117 suppressed the migration and invasion, induced apoptosis, reduced tumor growth and lung metastasis, and extended the survival rate in both in vitro and in vivo models of breast cancer. Overall, we demonstrated that ODZ10117 is a novel STAT3 inhibitor and may be a promising agent for the development of anticancer drugs.

## 1. Introduction

Signal transducer and activator of transcription 3 (STAT3) is a member of the STAT family consisting of seven proteins in mammals: STAT1, STAT2, STAT3, STAT4, STAT5A, STAT5B, and STAT6 [1,2]. It was first discovered independently as an acute-phase response factor that selectively binds to DNA in response to interleukin (IL)-6 and epidermal growth factor (EGF) [3,4]. STAT3 is a transcription factor that remains in the cytoplasm as an inactivated form and is expressed at a basal level under normal conditions. It can be activated by the phosphorylation of tyrosine 705 residue in response to cytokines, chemokines, and growth factors through receptor- and non-receptor-associated tyrosine kinases. Activated STAT3 can be translocated to the nucleus and regulates the transcription of a variety of genes involved in important biological functions, including cell proliferation, survival, differentiation, angiogenesis, tissue development, and immune responses. However, persistently activated STAT3 signaling is closely associated with oncogenic signaling and is frequently observed in numerous types of cancer cells and tumor tissues of cancer patients [1,5]. In fact, this signaling is positively correlated with cancer aggressiveness, malignancy, recurrence, drug resistance, and a poor prognosis via promoting the survival, proliferation, invasion, and metastasis of cancer cells and maintaining cancer stem cell (CSC) properties [6,7,8]. Therefore, targeting persistently activated STAT3 signaling is considered one of the important therapeutic options for cancer treatment.

Breast cancer is the most commonly diagnosed cancer and the second leading cause of cancer mortality in women worldwide. The incidence of breast cancer increases with age and the survival rate of patients generally decreases in case of invasive malignant characteristics [9]. Based on the receptor patterns of genomic expression profiling, breast cancer is classified into four major classes: luminal A, luminal B, human epidermal growth factor receptor-2 (HER2)-positive, and triple-negative breast cancer (TNBC) [10,11]. Patients with luminal subtypes of breast cancer are typically treated with hormonal and/or HER2-targeted therapy, and the prognosis is generally excellent with a 10-year survival rate of over 95%. In contrast, HER2-positive and TNBC subtypes are invasive breast cancers that are commonly associated with a poorer prognosis, a higher rate of distant recurrence, and the shortest overall survival rate of all breast cancers. These subtypes of cancer are highly aggressive and have low sensitivity to typical endocrine therapies and the limited number of therapeutic options than other subtypes [12,13,14]. In fact, STAT3 is persistently activated in more than 40% of breast cancer patients. Particularly, high level of activated STAT3 is observed in TNBC subtype, which is closely related to the promotion of cancer progression and metastasis, increased resistance to chemotherapy, and reduced overall survival rates in cancer patients [15,16,17,18]. Putting these evidences together, STAT3 is an important therapeutic target for cancer treatment. In this study, we identified 3-(2,4-dichloro-phenoxymethyl)-5-trichloromethyl-[1,2,4]oxadiazole (ODZ10117) as a novel STAT3 inhibitor and examined the anticancer activity of ODZ10117 in models of breast cancer.

## 2. Materials and Methods

### 2.1. Structure-Based Computational Database Screening

To discover small molecules that target the SH2 domain of STAT3, we prepared a 3D-structure of the SH2 domain by extracting the corresponding region from the X-ray structure of mouse STAT3 (mSTAT3), which was available from the protein data bank (PDB), entry 1BG1 [19]. Multiple sequence alignment between mSTAT3 and human STAT3 (hSTAT3) using the Clustal Omega server (https://www.ebi.ac.uk/Tools/msa/clustalo/, Windows 1.2.2) showed that the template and target sequence share 99.83% sequence identity and the remaining variability occurs in the N-terminal region. Amino acid sequences for mSTAT3 and hSTAT3 were downloaded from the UniProtKB database using the accession numbers P42227 and P40763, respectively. We used the mSTAT3 3D-structure for docking and the structure was prepared at default level using the Protein Preparation Wizard [20] in the Maestro utility of the Schrödinger 2017-4 Suites package. The chemical databases were obtained from our in-house library with a molecular weight of less than 300 g/mol and each compound was sketched using the ChemDraw Professional 16 software (16.0.1.4) and imported to the Maestro LigPrep module [21]. The default settings of the LigPrep module were used to prepare the ligands. The peptide Pro-pTyr-Leu was obtained from the 1BG1 protein and assigned a grid that covered 5Å surrounding Pro-pTyr-Leu. The Glide module [22] with the standard precision (SP) docking algorithm was used to further study docking and obtain docking solutions. Fifty docking poses were generated for each ligand and ranked according to Glide docking score. Tighter binding is reflected by a greater negative docking score and vice versa. We repeated the same procedures to dock the known STAT3 inhibitors S3I-201 [23] and STA-21 [24] against the SH2 domain of STAT3 for comparison. Figures were generated using the Discovery Studio Client 2018, and Pymol 2.1.0.

### 2.2. Cell-Based High-Throughput Screening

To perform cell-based high-throughput screening, we generated MDA-MB-231/STAT3-Luc cells stably expressing both the p21×STAT3-firefly luciferase [25] and pRL-TK Renilla luciferase reporter constructs. Cells were incubated for 24 h in the presence of each compound and the reporter activity was quantified by measuring the relative luciferase units. The luciferase activity was calculated using the ratio of the activity of firefly luciferase to that of Renilla luciferase. We also generated S2-NP/STAT92E-Luc cells stably expressing both the p10×STAT92E-firefly luciferase and RNA polymerase III Renilla luciferase reporter constructs, and a cell-based luciferase assay was performed as previously described [26].

### 2.3. Reagents

The known STAT3 inhibitors S3I-201 [23], STA-21 [24], and nifuroxazide [27] were purchased from Abcam and Sigma-Aldrich. Napabucasin, an inhibitor of STAT3 and cancer stemness [28], was purchase from MedChemExpress and the pan-JAK inhibitor AG-490 was purchased from Sigma-Aldrich. Recombinant human IL-6 was obtained from PROSPEC. Antibodies specific for phospho-JAK1 (Tyr1022/1023), JAK1, phospho-JAK2 (Tyr1007/1008), JAK2, phospho-JAK3 (Tyr980/981), phospho-TYK2 (Tyr1054/1055), TYK2, phospho-STAT1 (Tyr701), STAT1, phospho-STAT3 (Tyr705), phospho-STAT5 (Tyr694), phospho-STAT6 (Tyr641), STAT6, phospho-Akt (Ser473), Akt, phospho-Lyn (Tyr507), Lyn, phospho-Src (Tyr416), Src, phospho-ERK1/2 (Thr202/Tyr204), ERK1/2, PARP, caspase-3, Bcl-2, Bcl-xL, Mcl-1, survivin, cyclin D1, and active caspase 3 were purchased from Cell Signaling Technology. Antibodies specific for JAK3, STAT3, and STAT5 were obtained from Santa Cruz Biotechnology, and TWIST, pro/active MMP-2, and GAPDH were obtained from Abcam, R&D Systems and Abclone, respectively.

### 2.4. Cell Lines and Culture Conditions

MCF-10A cells were obtained from the American Type Culture Collection (ATCC) and maintained in DMEM/F-12 medium supplemented with 5% horse serum, 100 ng/mL cholera toxin, 20 ng/mL EGF, 0.5 mg/mL hydrocortisone, 10 μg/mL insulin, and 1% penicillin/streptomycin. Hs578T, MCF-7, MDA-MB-231, MDA-MB-468, T47D, ZR-75-1, and 4T1 cells were obtained from ATCC and maintained in DMEM, EMEM, or Leibovitz’s L-15 medium supplemented with 10% FBS and 1% penicillin/streptomycin. TUBO cells were maintained in DMEM supplemented with 10% FBS, 10% NCTC-109 medium, 2 mmol/L L-glutamine, 0.1 mmol/L MEM nonessential amino acids, and 1% penicillin/streptomycin. HDLM-2 and L540 cells were obtained from the German Collection of Microorganisms and Cell Cultures (DSMZ) and maintained in RPMI supplemented with 20% FBS and 1% penicillin/streptomycin. Other cancer cells were obtained from ATCC or DSMZ and maintained in DMEM, RPMI, or Leibovitz’s L-15 medium supplemented with 10% FBS and 1% penicillin/streptomycin or in accordance with the depositor’s specifications. MDA-MB-231/STAT3-Luc cells were maintained in Leibovitz’s L-15 medium supplemented with 10% FBS, 1% penicillin/streptomycin, and 500 μg/mL geneticin. All the cells were maintained at 37 °C in a humidified incubator containing 5% CO_2_. S2-NP cells were maintained in Schneider’s Drosophila medium supplemented with 10% FBS and 1% penicillin/streptomycin in an incubator at 25 °C. S2-NP/STAT92E-Luc cells were also maintained in the same medium as parental cells, but supplemented with 500 μg/mL geneticin.

### 2.5. Protein Extraction and Immunoblot Analysis

Protein samples were prepared on ice for 30 min using Triton X-100 lysis buffer [50 mM Tris-HCl (pH 7.4), 350 mM NaCl, 1% Triton X-100, 0.5% Nonidet P-40, 10% glycerol, 0.1% SDS, 1 mM EDTA, 1 mM EGTA, and 1 mM Na_3_VO_4_] containing 1 mM phenylmethylsulphonyl fluoride and protease and phosphatase inhibitor cocktails. Equal amounts of proteins were loaded on SDS-PAGE gels, separated by electrophoresis and transferred onto nitrocellulose membranes. The membrane was blocked in a blocking buffer [5% non-fat milk in 20 mM Tris-HCl (pH 7.5), 150 mM NaCl, and 0.1% Tween 20] and subsequently probed with corresponding primary antibodies at 4 °C overnight. Signals were detected using an ECL reagent, followed by incubation with horseradish peroxidase-conjugated corresponding secondary antibodies.

### 2.6. RNA Extraction, cDNA Preparation, and Quantitative Real-time PCR

Total RNA was extracted using TRIzol reagent and cDNA was synthesized using a ReverTra Ace^®^ qPCR RT Kit. Quantitative real-time PCR (qPCR) was performed using a QuantiFast SYBR Green PCR master mix with an Applied Biosystems 7300 thermocycler. Data were analyzed using comparative Ct quantification and the value for each sample was normalized to the value for the *GAPDH* gene. The primers used in this experiment were BCL-2 (QT00025011), BCL-XL (QT00236712), MCL-1 (QT00094122), SURVIVIN (QT01679664), MMP-2 (QT00088396), MMP-9 (QT00040040), TWIST (QT00011956), and GAPDH (QT0007924) were all obtained from Qiagen.

### 2.7. Immunoprecipitation

MDA-MB-231 cells were transfected with either Flag- or HA-tagged STAT3 plasmid, and whole-cell lysates were prepared on ice for 30 min in lysis buffer [25 mM HEPES (pH 7.7), 0.4 M NaCl, 1.5 mM MgCl_2_, 2 mM EDTA, 1% Triton X-100, and 0.5 mM DTT] containing protease and phosphatase inhibitor cocktails. The lysates were incubated with either anti-Flag or anti-HA antibody at 4 °C overnight, and the immune complexes were precipitated with protein G-Sepharose at 4 °C for 2 h. The immune complexes were separated by SDS-PAGE and probed with antibodies specific for tyrosine phosphorylated STAT3 (pY^705^-STAT3), STAT3, Flag, and HA antibodies. Antibodies specific for Flag and HA were obtained from Cell Signaling Technology and Abcam, respectively.

### 2.8. Immunofluorescence

MDA-MB-231 cells grown in lysine-coated 24-well plates were fixed for 45 min at room temperature in 3% paraformaldehyde in PBS and permeabilized for 10 min with 0.1% Triton X-100 in PBS. The plates were then blocked for 20 min with 3% BSA in PBS and incubated with pY^705^-STAT3 antibody at 4 °C overnight. After washing with PBS, the dishes were incubated with fluorescein isothiocyanate (FITC)-conjugated secondary antibody at room temperature for 2 h. Nuclei were counterstained with 4′,6-diamidino-2-phenylindole (DAPI) and images were captured using a Zeiss Axiovert 200 inverted fluorescence microscope (Oberkochen, Germany) with an LSM 510 META system (ZEN 2011).

### 2.9. Immunohistochemistry

Tissues were fixed with 4% paraformaldehyde in 0.1 M phosphate buffer (pH 7.4) and embedded in paraffin. The paraffin blocks were cut in 4-μm thick sections and the sections were mounted on glass slides, dewaxed, rehydrated with grade ethanol, and then stained with hematoxylin and eosin (H&E). To perform immunohistochemical analyses, rehydrated slide sections were quenched with endogenous peroxidase for 10 min in 3% hydrogen peroxide, blocked for 30 min in PBS containing 10% goat serum at room temperature, and then incubated with corresponding primary antibody overnight at 4 °C. After washing, the sections were incubated with biotinylated secondary antibody compatible with the primary antibody for 30 min, subsequently incubated with streptavidin-HRP for 40 min, and then stained with 3,3′-diaminobenzidine (DAB). Digital images were obtained using the LAS microscope software (Wetzlar, Germany).

### 2.10. Cell Viability Assay

Cells were seeded at a density of 1 × 10^4^ cells per well in a 96-well plate and incubated in culture medium until 70–80% confluence. The cells were further incubated for 24 h with either vehicle alone or various concentrations of ODZ10117. Cell viability was determined at 450 nm using a microplate reader after further incubation for 2–4 h at 37 °C, followed by the addition of 10 μL EZ-CyTox enhanced cell viability assay reagent.

### 2.11. Migration and Invasion Assays

The migration assay was performed on MDA-MB-231 cells when they reached greater than 90% confluence. Cells were incubated for 24 h with freshly prepared Leibovitz’s L-15 medium containing either vehicle alone or ODZ10117, followed by scratching with pipette tips and washing with PBS. The images were obtained using the LAS microscope software.

The invasion assay was performed using a Boyden chamber system containing growth factor reduced Matrigel diluted with serum-free media at a ratio of 1:3. Diluted Matrigel was transferred into a 24-transwell support (BD 24-well insert, 8 μm pore transparent PET filter) and incubated at 37 °C for 4–5 h for gelling. MDA-MB-231 cells in 100 μL Leibovitz’s L-15 medium containing 1% FBS were seeded in the upper chamber and incubated for 24 h in the presence of either vehicle alone or ODZ10117. The lower chamber was filled with 500 μL of 10% Leibovitz’s L-15 medium containing 5 μg/mL fibronectin. The Matrigel-containing upper chamber was rinsed with PBS, fixed, stained with Diff-Quik solution, and subsequently rinsed with distilled water. The migrated cells were captured using the LAS microscope software.

### 2.12. Flow Cytometry

To analyze cell cycle and apoptotic cell populations, cells were fixed with 70% ice-cold ethanol, washed with PBS, incubated with 50 μg RNase at 37 °C for 1 h, and then stained with 20 μg propidium iodide (PI) at 4 °C in the dark. For Annexin V staining, Annexin V binding buffer containing FITC conjugated with anti-Annexin V antibody was used according to the manufacturer’s protocol. PI and/or Annexin V stained cells were counted by flow cytometry using the BD LSRFortessa^TM^ cell analyzer (San Jose, CA, USA).

### 2.13. Bioluminescence Imaging

Mice were intraperitoneally administered firefly D-Luciferin potassium salt at a dose of 150 mg/kg body weight in Dulbecco’s PBS. Bioluminescence images were obtained with the IVIS Lumina system under anesthesia with 2% isoflurane. Analysis was performed using Living Image^®^ Software (Windows 4.7.3) by measuring the photon flux (photons/s/cm^2^/sr) for approximately 1 h using a region of interest manually drawn over the body of the mouse.

### 2.14. Mouse Xenograft Studies

An orthotopic xenograft model was generated by the introduction of MDA-MB-231 cells (1 × 10^6^ cells in 50 μL HBSS containing growth factor reduced 25% Matrigel) into the right fourth mammary fat pad of 6-week-old female BALB/c nude mice under anesthesia using a 30-gauge insulin needle (n = 6). Intraperitoneal treatment with vehicle control (5% EtOH, 40% PEG400, 55% DW) or ODZ10117 (1 mg/kg or 10 mg/kg) was initiated at 17 days post-tumor cell inoculation, followed by injecting 5 times per week for 23 days.

To generate syngeneic xenograft model, 4T1-Luc cells (5 × 10^5^ cells in 50 μL HBSS containing growth factor reduced 25% Matrigel) were injected into the right fourth mammary fat pad of 6-week-old female BALB/c mice using a 30-gauge insulin needle under anesthesia. After 11 days, mice were randomly divided into three groups (*n* = 10) and vehicle (DMSO and corn oil = 1:9) alone, ODZ10117 (1 or 10 mg/kg), or napabucasin (10 mg/kg) was intraperitoneally administered 5 times per week for 3 weeks. At the end of the experiment, the lungs were resected and fixed in Bouin’s solution and the visible metastatic nodules in the lungs were quantified.

We also established subcutaneous xenograft model by injection of MDA-MB-231 cells (2.5 × 10^4^ cells in 100 μL PBS containing growth factor reduced 25% Matrigel) into the neck of 6-week-old female BALB/c nude mice. Mice were randomly divided into two groups (*n* = 6) after 14 days and intratumorally injected with vehicle (0.1% DMSO) alone or ODZ10117 (10 mg/kg) at a two-day interval for 2 weeks. The tumor sizes were measured by a caliper ruler at two-day intervals and tumor volumes were calculated using *a* × *b*^2^/2 (where *a* is the width at the widest point of the tumor and *b* is the width perpendicular to *a*). All surgical and experimental procedures were approved by the Institutional Animal Care and Use Committee at the Daegu-Gyeongbuk Medical Innovation Foundation (DGMIF) and Seoul National University College of Medicine, and were performed in accordance with government and institutional guidelines and regulations.

### 2.15. Statistical Analysis

Statistical analyses were performed using GraphPad Prism 5.0. Data are represented as mean ± standard error of mean (SEM). Significance was determined using Student’s *t*-test and values of *p* < 0.05 were considered statistically significant. Survival analysis was performed by the Kaplan–Meier method using a log-rank test.

## 3. Results

### 3.1. Subsection

#### 3.1.1. Identification of ODZ17690 as a Hit Compound by Targeting the SH2 Domain of STAT3

To identify small-molecule inhibitors that target STAT3, we performed combined screening assays in combination with structure-based computational database screening and cell-based high-throughput screening using our in-house compound library with molecular weight less than 300 g/mol. For virtual screening, we used the 3D structure of the SH2 domain of STAT3 (PDB ID: 1BG1) and selected the coordinate from the complex structure of STAT3-DNA (PDB: 1BG1) [19]. The docking was performed using the Glide module [22] with the SP docking algorithm. We also performed cell-based high-throughput screening using the luciferase reporter constructed S2-NP/STAT92E-Luc and MDA-MB-231/STAT3-Luc cells.

We identified 5-*tert*-butyl-3-(3-nitro-phenoxymethyl)-[1,2,4]oxadiazole (ODZ17690) as a hit compound, which showed the lowest Glide docking score at −3.37 kcal/mol. The nitro group of ODZ17690 docked in the polar environment of the SH2 domain and interacted with Lys591 and Arg609 via a salt bridge and attractive charge interaction. Hydrogen bond interactions were shown by the nitro group and oxadiazole with the Ser611 and Glu638 residues, respectively. Pro639 was involved in a pi-alkyl interaction with the phenyl group of ODZ17690. Glu612, Ser613, Thr620, Trp623, Lys626, and Gln635 were involved in van der Waals interactions with ODZ17690 (Supplementary Figure S1). ODZ17690 inhibited the transcriptional activity of STAT92E and STAT3 in Upd- or Hop*^tum-l^*-induced S2-NP/STAT92E-Luc cells and MDA-MB-231/STAT3-Luc cells without affecting their viability (Supplementary Figure S2A–C). In particular, ODZ17690 specifically inhibited the tyrosine phosphorylation of STAT3, but not that of other STAT and JAK family proteins in human Hodgkin’s lymphoma cells (Supplementary Figure S2D,E). These results indicate that ODZ17690 is particularly well targeted to STAT3.

#### 3.1.2. Discovery of ODZ10117 as a STAT3 Inhibitor

**Figure 1:**
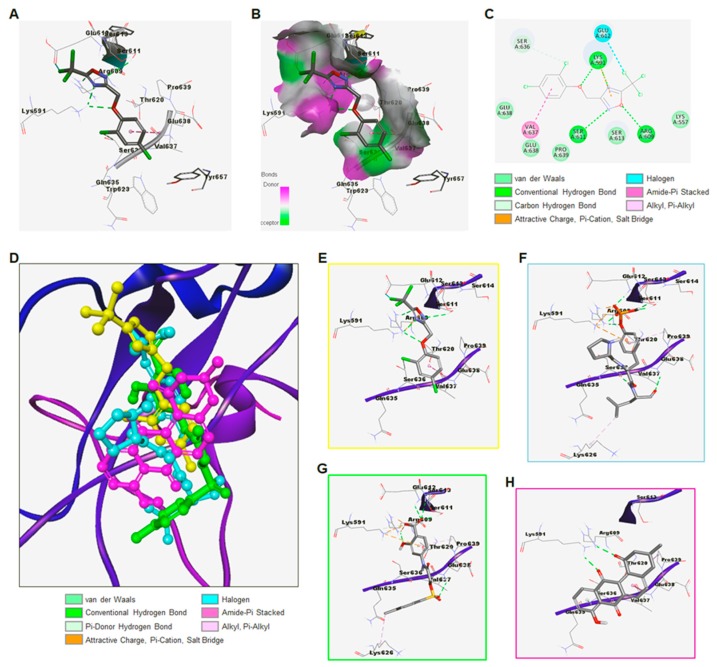
Molecular docking of ODZ10117 against the SH2 domain of STAT3. (**A**) Docked model of ODZ10117 is shown in stick and the surrounding residues of the SH2 domain of STAT3 are shown by a line model. Hydrogen bonds are shown for Lys591, Arg609, and Ser611. Halogen bond between Cl and Glu612 is shown by a cyan dash. (**B**) Transparent hydrogen bond acceptor/donor surface is shown for surrounding residues. (**C**) 2D-interaction plot is shown and color-coded by various interaction types. (**D**) Superimposed docked models of ODZ10117 (yellow), Pro-pTyr-Leu (cyan), S3I-201 (green), and STA-21 (magenta) over STAT3. (E-H) Docked models of ODZ10117 (**E**), Pro-pTyr-Leu (**F**), S3I-201 (**G**), and STA-21 (**H**) surrounding 4Å residues of STAT3. Color codes for the different types of interactions are shown at the bottom of D.

To increase the pharmacological activity, we synthesized 144 derivatives that were based on the core structure of ODZ17690 and finally selected ODZ10117 from the results of combined screening assays and immunoblot analyses (Supplementary Figure S3). The molecular docking results showed that ODZ10117 snugly fits into the phospho-tyrosine binding pocket on the SH2 domain of STAT3 with a docking score of −6.17 kcal/mol and participated in many interactions with surrounding residues of the SH2 domain. ODZ10117 mainly showed hydrogen bond interactions with Lys591, Arg609, and Ser611. It also displayed an amide-pi interaction with Val637. Chlorine from the tri-chloromethyl group showed a halogen bond interaction with Glu612. Other interactions were mainly of the van der Waals type (Figure 1A–C,E).

To perform a comparative analysis of ODZ10117 with tripeptide Pro-pTyr-Leu as a substrate and reference compounds S3I-201 and STA-21 as a STAT3 inhibitor by binding to the site of SH2 dimerization [23,24], we created superimposed orientations of the selected ligand postures (Figure 1D). First, we docked a tripeptide in the SH2 domain and analyzed the docking orientations of the tripeptide. The results suggest that the best docking orientation of the tripeptide had a 1. 97Å root-mean-square deviation (RMSD) with the native tripeptide and docking score of −6.43 kcal/mol. The tripeptide participated in several interactions with the surrounding SH2 domain residues. The phospho-tyrosine of the tripeptide showed pi-cation and salt bridge interactions with the crucial Lys591. Pro639 showed a pi-alkyl interaction with the pTyr. The phosphate group showed several hydrogen bond interactions with the Lys591, Arg609, Ser611, the main chain of Glu612 and Ser613. Also, Arg609 showed an attractive charge type interaction with the phosphate O. Leu706 of the tripeptide showed hydrogen bond interactions with the Ser636 and Glu638, and an alkyl-alkyl interaction with the side chain of Lys626. The strong network of interaction patterns led to the higher docking score of the tripeptide inside the SH2 domain of STAT3 (Figure 1F and Supplementary Figure S4A).

**Figure 2:**
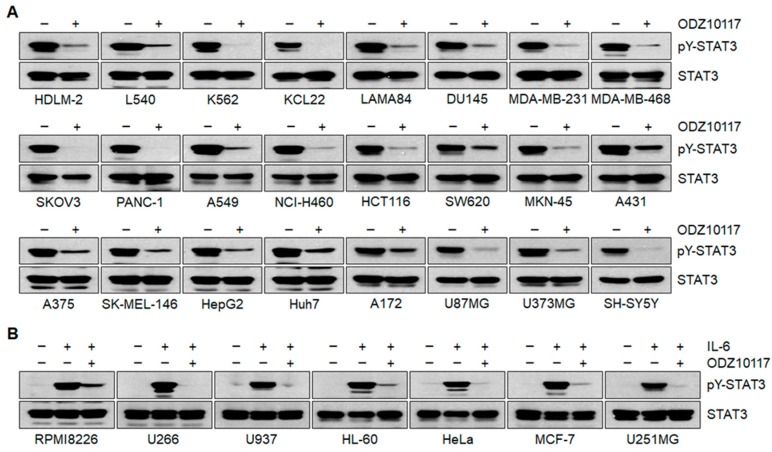
ODZ10117 inhibits tyrosine phosphorylation of STAT3 in various human cancer cell lines. (**A**,**B**) Various types of human cancer cell lines with STAT3 constitutively activated (**A**) or activated by IL-6-stimulation (20 ng/mL) for 10 min (**B**) were incubated for 24 h with either vehicle (0.1% DMSO) alone or ODZ10117 (40 μM) and immunoblotting was performed.

The S3I-201 was docked inside the SH2 domain with a Glide docking score of −5.87 kcal/mol. The carboxylic acid group of S3I-201 was bound to Arg609, Ser611, Glu612, and Ser613 via hydrogen bond interactions. The hydroxyl group on the phenyl ring also hydrogen bonded with Arg609. Another hydrogen bond was observed between the Glu638 and sulphonyl O of S3I-201. Lys626 showed an alkyl interaction with the 4-methylphenyl, and Pro639 displayed a pi-alkyl interaction with the 4-carboxyphenyl moiety. Thr620, Trp623, Gln635, Ser636, and Val637 interacted through van der Waals interactions with S3I-201 (Figure 1G and Supplementary Figure S4B). The STA-21 bound with a Glide docking score of −3.58 kcal/mol, and showed two hydrogen bond interactions with the crucial Lys591. Amide-pi and alkyl interactions were observed between STA-21 and Val637 and Pro639. Pi-donor hydrogen bond interactions were observed between the pi-cloud of the fused ring system of STA-21 and main chain NH of Ser636. This smaller ligand size led to a minimal interaction network with the surrounding SH2 domain of STAT3 (Figure 1H and Supplementary Figure S4C). These results indicate that ODZ10117 is more effective than ODZ17690, and the binding of ODZ10117 to the SH domain of STAT3 was comparable to that of the tripeptide and lower than those for S3I-201 and STA-21, indicating higher affinity. We further observed that ODZ10117 effectively inhibited the level of tyrosine phosphorylated STAT3 in various cancer cell lines that are persistently activated or activated by IL-6-stimulation (Figure 2).

#### 3.1.3. Synthesis of ODZ10117

**Figure 3:**
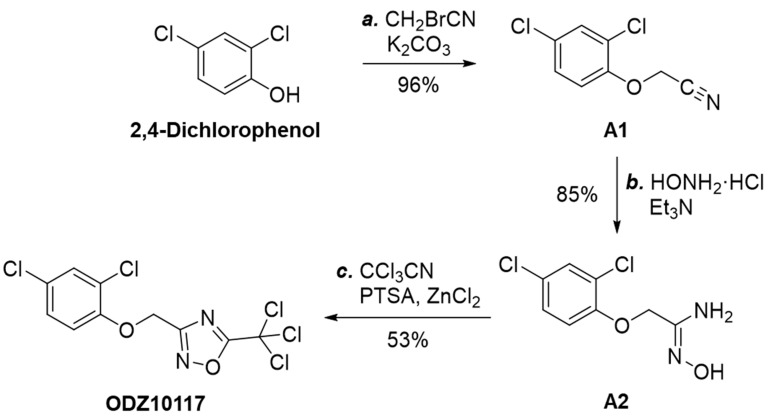
Synthetic scheme of ODZ10117. Reagents and conditions: (**a**) bromoacetonitrile (1.0 equiv), K_2_CO_3_ (1.0 equiv), DMF, room temperature, 5 h, 96%; (**b**) hydroxylamine hydrochloride (1.3 equiv), Et_3_N (1.3 equiv), EtOH:H_2_O (9:1), 100°C, 1 h, 85%; (**c)** trichloroacetonitrile (1.0 equiv), *p*-toluenesulfonic acid monohydrate (0.5 equiv), zinc chloride (0.5 equiv), DMF, 80°C, 16 h, 53%.

We synthesized ODZ10117 to further investigate its pharmacological efficacy in both in vitro and in vivo models of breast cancer. ODZ10117 was readily synthesized from commercial 2,4-dichlorophenol in three steps (Figure 3). Alkylation of 2,4-dichlorophenol with bromoacetonitrile in the presence of potassium carbonate produced 2-(2,4-dichlorophenoxy)acetonitrile (A1) [29] in excellent yield. Treatment of this nitrile with hydroxylamine [30] yielded 2-(2,4-dichlorophenoxy)-*N*′-hydroxyacetimidamide (A2). The reaction of 2-(2,4-dichlorophenoxy)-*N*′-hydroxyacetimidamide with trichloroacetonitrile was catalyzed by *p*-toluenesulfonic acid/zinc chloride [31] to give the 1,2,4-oxadiazole heterocycle ODZ10117 in modest yield. The ^1^H and ^13^C NMR spectra of 2-(2,4-dichlorophenoxy)acetonitrile, 2-(2,4-dichlorophenoxy)-*N*′-hydroxyacetimidamide, and ODZ10117 are shown in Supplementary Figure S5–S7. Detailed synthetic information was described in Supplementary Results.

#### 3.1.4. ODZ10117 Inhibits STAT3 Signaling In Vitro

**Figure 4:**
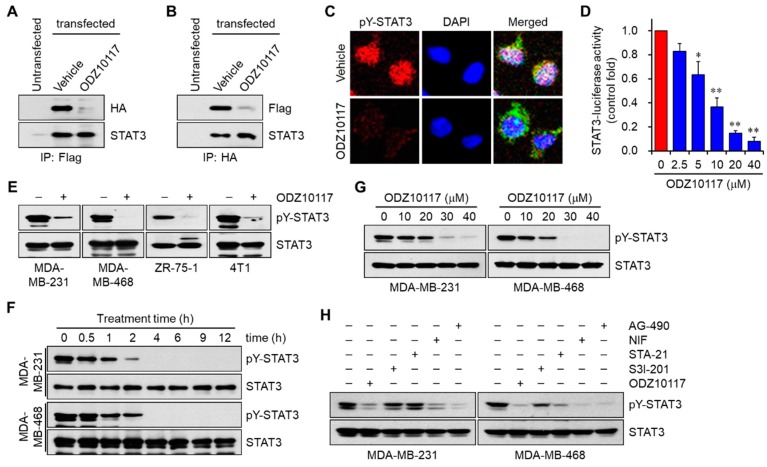
ODZ10117 inhibits STAT3 activation. (**A**,**B**) MDA-MB-231 cells were transfected with either Flag- or HA-tagged STAT3 plasmid, incubated for 24 h with either vehicle (0.1% DMSO) alone or ODZ10117 (40 μM) and then performed immunoprecipitation assay. (**C**) MDA-MB-231 cells were incubated for 24 h with either vehicle (0.1% DMSO) alone or ODZ10117 (40 μM) and then performed immunofluorescence staining. Nuclei were counterstained with DAPI. (**D**) MDA-MB-231/STAT3-Luc cells were incubated for 24 h with various concentrations of ODZ10117, and STAT3-reporter activity was determined. Data are represented as mean ± SEM of three independent experiments. * *p* < 0.05 and ** *p* < 0.005 compared to the vehicle-treated group. (**E**–**G**) Cells were treated with ODZ10117 (40 μM) for 24 h (**E**), or treated with ODZ10117 (40 μM) in a time-dependent manner (**F**) or for 9 h in a concentration-dependent manner (**G**), and then performed immunoblot analysis. (**H**) Comparison on the effects of ODZ10117 (40 μM) and the known STAT3 inhibitors S3I-201 (100 μM), STA-21 (100 μM), nifuroxazide (NIF, 100 μM), and AG-490 (150 μM) on tyrosine phosphorylation of STAT3. Cells were incubated for 9 h with each compound and then performed immunoblot analysis.

According to the STAT3 signaling cascades, tyrosine phosphorylated STAT3 sequentially results in the homodimerization, nuclear translocation, and transcriptional activation of STAT3, inducing the expression of numerous target genes by binding to specific DNA sequences [32]. The binding of ODZ10117 to the SH2 domain of STAT3 is thought to result in the breakdown of these sequential cascades. To determine the effect of ODZ10117 on STAT3 dimerization, STAT3 was overexpressed in MDA-MB-231 cells by transfection with either Flag- or HA-tagged STAT3 plasmid. STAT3 overexpression resulted in a dramatic increase in the levels of both total and tyrosine-phosphorylated STAT3, and the phosphorylated form was decreased by ODZ10117 without altering the level of total STAT3 (Supplementary Figure S8). Additionally, ODZ10117 significantly decreased the homodimerization of STAT3 compared to that in the vehicle-treated group (Figure 4A,B). Furthermore, ODZ10117 decreased the nuclear translocation of STAT3 (Figure 4C), and inhibited the transcriptional activity of STAT3 in a concentration-dependent manner with an IC_50_ value of 7.5 μM (Figure 4D). These results clearly indicate that ODZ10117 is an effective STAT3 inhibitor.

#### 3.1.5. ODZ10117 has a Greater Inhibition on STAT3 Activation than the Known Inhibitors

We further verified whether ODZ10117 can inhibit tyrosine phosphorylation of STAT3 in breast cancer cells. ODZ10117 treatment resulted in a dramatic decrease in the level of tyrosine-phosphorylated STAT3 in various breast cancer cells (Figure 4E). In addition, the time- and concentration-dependent results showed that ODZ10117 effectively decreased the level of tyrosine-phosphorylated STAT3 following incubation over 2 h at 40 μM (Figure 4F and Supplementary Figure S9A) or for 9 h over 20 μM (Figure 4G and Supplementary Figure S9B). We further determined the inhibitory activity of ODZ10117 on tyrosine-phosphorylated STAT3 in comparison with the known STAT3 inhibitors S3I-201 [23], STA-21 [24], and nifuroxazide [27], and pan-JAK inhibitor AG-490. The inhibitory effect of ODZ10117 on tyrosine-phosphorylated STAT3 was greater than the known inhibitors in breast cancer cells that are persistently activated or activated by IL-6-stimulation (Figure 4H and Supplementary Figure S9C–E). The above-mentioned results indicate that ODZ10117 is an effective STAT3 inhibitor that directly targets the SH2 domain of STAT3, and has a greater inhibitory efficacy than other known inhibitors such as S3I-201, STA-21, nifuroxazide, and AG-490.

#### 3.1.6. ODZ10117 Does Not Affect Other STAT Family Proteins and Upstream Regulators of STAT3

**Figure 5:**
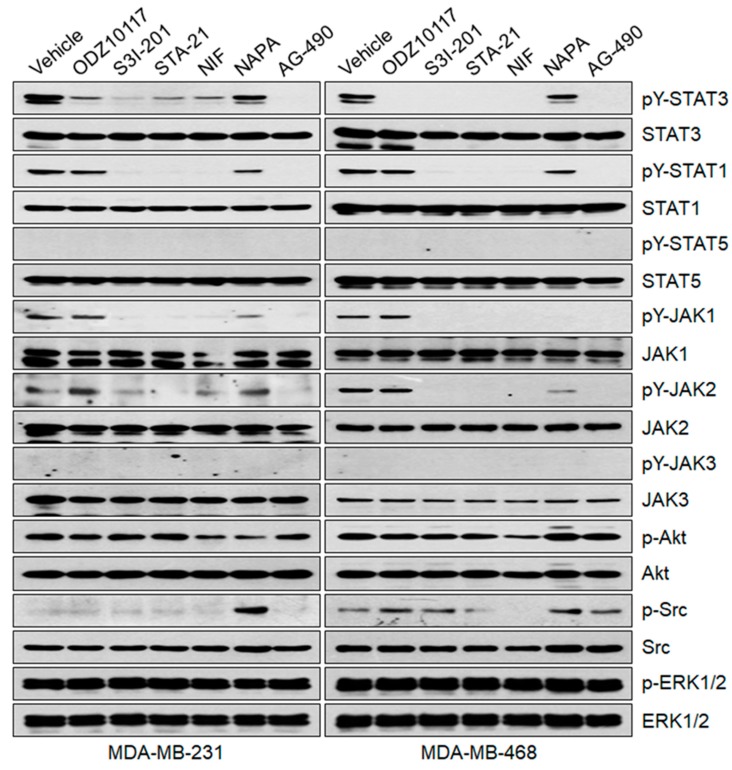
ODZ10117 does not affect other STAT family members and upstream regulators of STAT3. Cells were incubated for 16 h with vehicle (0.1% DMSO) alone, ODZ10117 (ODZ, 40 μM) or the known STAT3 inhibitors S3I-201 (100 μM), STA-21 (100 μM), nifuroxazide (NIF, 100 μM), napabucasin (NAPA, 4 μM), or AG-490 (150 μM), and then performed immunoblot analysis.

The mammalian STAT family shares structural similarities and the family proteins can be activated by several upstream regulators including mainly receptor and non-receptor tyrosine kinases [33]. To determine the specificity of ODZ10117 for STAT3, we verified whether ODZ10117 affects other STAT family proteins and upstream regulators of STAT3. Although ODZ10117 inhibited the tyrosine phosphorylation of STAT3, it did not significantly affect other STAT family proteins, including STAT1, STAT5, and STAT6, in breast cancer cells and Hodgkin’s lymphoma cells (Figure 5 and Supplementary Figure S10A).

Next, we evaluated the JAK family of tyrosine kinases, which are direct upstream regulators of STAT family proteins. ODZ10117 did not significantly inhibit the JAK family of tyrosine kinases, including JAK1, JAK2, JAK3, and TYK2, in breast cancer cells and Hodgkin’s lymphoma cells (Figure 5 and Supplementary Figure S10B). ODZ10117 also did not significantly affect other upstream regulators of STAT3, including serine/threonine protein kinase Akt, the Src family tyrosine kinases Src and Lyn, and MAP kinases ERK1/2 (Figure 5 and Supplementary Figure S10B). The pan-JAK inhibitor AG-490 effectively inhibited the activation of various JAK, STAT, and Src family proteins. Interestingly, although the effect was cell line-dependent, other STAT3 inhibitors, including S3I-201 [23], STA-21 [24], nifuroxazide [27], and napabucasin [28], inhibited the tyrosine phosphorylation of various STAT and JAK family proteins, indicating that these compounds are not STAT3-specific inhibitors. Collectively, these results indicate that ODZ10117 is a novel STAT3-specific inhibitor.

#### 3.1.7. ODZ10117 Decreases Cell Viability by Inducing Apoptosis

**Figure 6:**
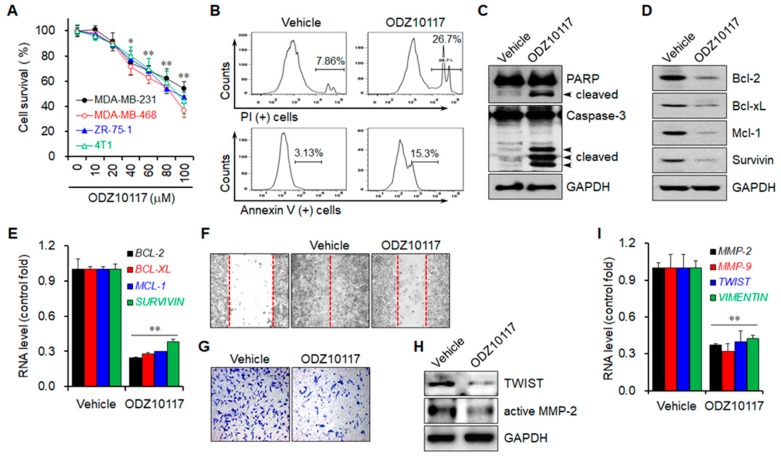
ODZ10117 induces apoptotic cell death and decreases migration and invasion of breast cancer. (**A**) Cells were incubated for 24 h with various concentrations of ODZ10117 and cell viability was determined. (**B**–**E**) MDA-MB-231 cells were incubated for 24 h with either vehicle (0.1% DMSO) alone or ODZ10117 (40 μM), and then performed FACS (**B**), immunoblot (**C**,**D**), and qPCR (**E**) analyses. 2D graphs indicate PI- (upper) or Annexin V-positive (bottom) cells. (**F**–**I**) MDA-MB-231 cells were incubated for 24 h with either vehicle (0.1% DMSO) alone or ODZ10117 (40 μM), and then performed wound healing (**F**) and Matrigel invasion (**G**) assays or immunoblot (**H**) and qPCR (**I**) analyses. Magnification, 200×. GAPDH served as a loading control. Data are represented as mean ± SEM of three independent experiments. * *p* < 0.05 and ** *p* < 0.005 compared to the vehicle-treated group.

Persistently activated STAT3 is associated with excessive cell proliferation and survival in cancer cells [6,8], indicating that targeting STAT3 can decrease these properties of cancer cells. ODZ10117 treatment decreased the viability of breast cancer cells in a concentration-dependent manner (Figure 6A). The results of FACS analyses showed that ODZ10117 increased the population of dead cells by more than three-fold, and among the dead cells, apoptotic cell death was increased by around five-fold by ODZ10117 (Figure 6B). In addition, ODZ10117 treatment increased the fragmentation of both PARP and caspase-3 (Figure 6C) and downregulated the mRNA and protein levels of anti-apoptotic genes such Bcl-2, Bcl-xL, Mcl-1, and survivin (Figure 6D,E), which are regulated by STAT3. These results indicate that ODZ10117 decreased the survival of breast cancer cells by inducing apoptotic cell death via activation of apoptotic proteins and inhibition of anti-apoptotic gene expression.

#### 3.1.8. ODZ10117 Reduces the Migration and Invasion of Breast Cancer Cells

The migration and invasion of cancer cells into the bloodstream and surrounding tissues are critical steps in cancer metastasis, and the transcription of many target genes associated with these processes is regulated by STAT3 [34]. Therefore, we performed in vitro wound healing and Matrigel invasion assays to determine whether ODZ10117 affects the migration and invasion of breast cancer cells. ODZ10117 treatment reduced the migration and invasion of breast cancer cells compared to the vehicle-treated control group (Figure 6F,G). In addition, the protein levels of TWIST and active MMP-2 were decreased upon ODZ10117 treatment compared to those in the vehicle-treated control group (Figure 6H). Consistent results were observed concerning mRNA levels, with a reduction in STAT3-dependent target genes such as *MMP-2*, *MMP-9*, *TWIST*, and *VIMENTIN* in the presence of ODZ10117 (Figure 6I). These results suggest that ODZ10117 may suppress cancer metastasis by targeting STAT3.

#### 3.1.9. ODZ10117 Suppresses Tumor Growth in Breast Cancer Xenografts

**Figure 7:**
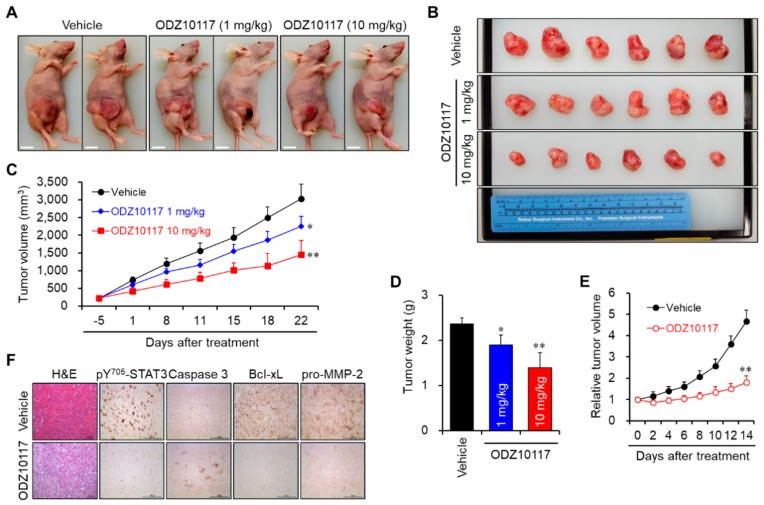
ODZ10117 suppressed tumor growth in breast cancer xenograft models. (**A**–**D**) Orthotopic xenograft model was generated by injection of MDA-MB-231 cells into the right fourth mammary fat pad of female BALB/c mice. The tumor growth (**A**), tumor size (**B**), tumor growth curve (**C**), and tumor weight (**D**) were represented. (**E**,**F**) Subcutaneous xenograft model was established by injecting MDA-MB-231 cells in suspension with 25% Matrigel-containing PBS into the neck of female BALB/c nu/nu nude mice. The tumor growth curve (**E**) and IHC analysis (**F**) were represented. * *p* < 0.05 and ** *p* < 0.005 compared to the vehicle-treated group.

Targeting STAT3 is a promising therapeutic strategy in many types of cancer patients. Therefore, we further investigated whether ODZ10117 suppresses tumor growth and metastasis in in vivo breast cancer xenograft models. To evaluate the in vivo pharmacological activity of ODZ10117, we first generated an orthotopic breast cancer xenograft model by injecting MDA-MB-231 cells into the right fourth mammary fat pad of BALB/c mice. At 17 days after inoculation, the tumor bearing mice were intraperitoneally injected with vehicle alone or ODZ10117 (1 mg/kg or 10 mg/kg) 5 times a week for 23 days. The administration of ODZ10117 suppressed the tumor growth compared with the vehicle-treated group (Figure 7A–D), without affecting the body weight (Supplementary Figure S11A).

We also generated subcutaneous xenograft model by injecting MDA-MB-231 cell suspensions in Matrigel-containing PBS into the neck of BALB/c nude mice. After 2 weeks of implantation, vehicle alone or ODZ10117 (10 mg/kg) was intratumorally injected into the tumor-bearing mice at 2-day intervals for 2 weeks. Tumor growth was dramatically increased in the vehicle-treated mice, whereas the administration of ODZ10117 significantly suppressed tumor growth (Figure 7E). Upon histological evaluation, the tumor population and the levels of active STAT3, Bcl-xL, and pro-MMP-2 were elevated, and that of active caspase-3 was decreased in the vehicle-treated mice, indicating that the cancer cells were actively growing in this group. However, the administration of ODZ10117 remarkably suppressed the tumor population and the levels of active STAT3, Bcl-xL, and pro-MMP-2, and increased the level of active caspase-3 (Figure 7F).

#### 3.1.10. ODZ10117 Suppresses Tumor Growth and Lung Metastasis in Breast Cancer Xenograft

**Figure 8:**
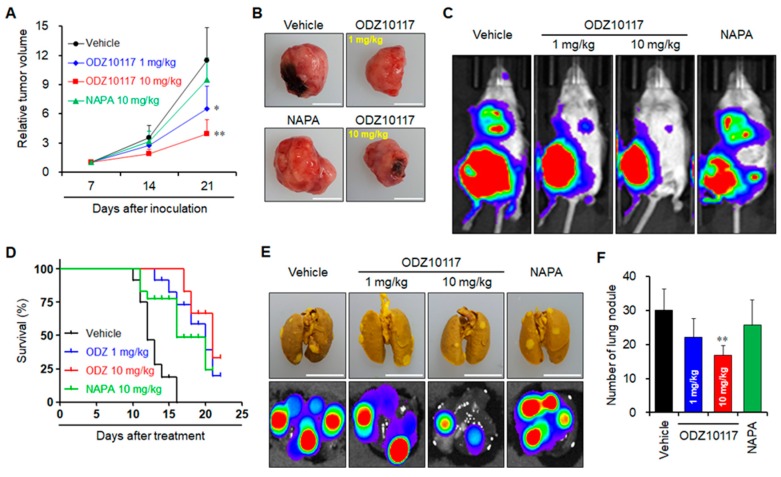
ODZ10117 inhibits tumor growth and lung metastasis and increases survival in breast cancer xenograft model. (**A**–**F**) Syngeneic breast metastasis xenograft model was generated by injection of 4T1-Luc cells into the right fourth mammary fat pad of female BALB/c mice. Primary tumor growth is shown (**A**,**B**). Representative bioluminescence images of whole body (**C**). Kaplan-Meier survival graph of tumor-bearing mice (**D**). Lung metastatic tumor nodules were observed on the surface (upper) and bioluminescence images (bottom) of the lung (**E**) and lung nodules were counted (**F**). * *p* < 0.05 and ** *p* < 0.005 compared to the vehicle-treated group.

We generated syngeneic xenograft model of spontaneous breast cancer metastasis by injecting 4T1 cells carrying the luciferase gene into the right fourth mammary fat pad of BALB/c mice. At 11 days after inoculation, the tumor-bearing mice were intraperitoneally injected with vehicle alone, ODZ10117 (1 mg/kg or 10 mg/kg), or napabucasin (10 mg/kg) 5 times a week for 3 weeks. The administration of ODZ10117 significantly reduced the progression of primary tumor growth compared with the vehicle-treated group (Figure 8A–C), and showed an excellent extension of median survival rates, which increased from 12 days to 20 days and 21 days, respectively (Figure 8D), without affecting the body weight (Supplementary Figure S11B). Additionally, ODZ10117 treatment reduced lung metastasis of tumor cells with tumor nodules in the lungs (Figure 8C,E,F). However, napaubcasin did not demonstrate significant suppression of tumor growth and metastasis, and the median survival rate was 16 days. Collectively, the results of in vivo xenografts models indicate that ODZ10117 exerted effective anticancer activity that suppressed tumor growth and metastasis, leading to the increased survival rate of the mice.

## 4. Discussion

Disrupted signaling pathways are generally associated with the development of many types of cancers [35,36], indicating that these signaling pathways can be promising therapeutic targets for cancer therapy and drug development. Among such pathways, aberrantly activated STAT3 signaling is considered an attractive therapeutic target for the treatment of many types of human diseases, including cancer [37,38]. In fact, elevated activation of STAT3 is observed in many types of solid and hematological cancers, and has recently been suggested as an important target molecule in the pre-therapeutic assessment of cancer patients [39]. In particular, breast cancer remains the second most common type of cancer, a primary cause of death in women, and one of the most expensive malignancies to treat [40]. The survival rate of breast cancer patients depends on the diagnostic timing and characteristics of cancer cells. Survival is lower when the cancer is diagnosed at a later stage and the cancer cells show higher metastatic potential and CSC traits. These cases of cancer are responsible for the difficulty in treating and important factors in determining the prognosis of breast cancer patients [9]. Specifically, higher level of tyrosine phosphorylated STAT3 was observed in TNBC subtype of breast cancer cells than those of other subtypes [15,16,17,18].

Many lines of compelling evidence support that hyperactivated STAT3 signaling is positively correlated with tumor progression, malignancy, recurrence, drug resistance, and a poor prognosis by increasing the metastatic potential and maintaining CSC properties of cancer cells [5,6,7,8,32,33]. Thus, targeting STAT3 may be an important strategy in cancer treatment. Targeted therapy is a recently developed type for cancer treatment that uses small-molecule drugs or monoclonal antibodies to more precisely target specific signaling molecules. This therapy has become a quickly growing part of the treatment plan for many types of cancer [40,41]. In the present study, we performed two-track screening assays in combination with structure-based computational database screening and cell-based high-throughput screening to identify small-molecule inhibitors for STAT3-targeted cancer therapy. Finally, we discovered ODZ10117 as a novel STAT3-specific inhibitor and determined its pharmacological activities in both in vitro and in vivo models of breast cancer.

The STAT family consists of seven proteins in mammals. Although the STAT family proteins are encoded by separate genes, they share a common set of structural domains that show functional similarities [33,42]. STAT3 can be activated in a paracrine manner by upstream regulators such as receptor- and non-receptor-associated kinases, including JAKs, Akt, Src family kinases, and MAP kinases [15,43]. Because the STAT family proteins and their upstream regulators can crosstalk with other signaling pathways associated with normal physiological functions, non-selective STAT3 inhibitors can influence normal physiological functions. For this reason, the development of selective STAT3 inhibitors has many impediments. To date, only a few STAT3-specific inhibitors have been developed [6,33,44,45], but the inhibitors have not yet been studied in clinical trials. Therefore, the discovery of small-molecules specifically targeting STAT3 is an important issue for treating human diseases caused by STAT3 signaling. Fortunately, ODZ10117 showed strong specificity for STAT3, regardless of other STAT family proteins and upstream regulators. Interestingly, we found that the known STAT3 inhibitors affected other STAT family proteins and upstream regulators, although they showed cell-line dependent effects, indicating that they are not STAT3-specific inhibitors. The STAT3-specificity and the STAT3 inhibitory effect of ODZ10117 were much stronger than the known STAT3 inhibitors such as S3I-201, STA-201, nifuroxazide, and napabucasin.

## 5. Conclusions

In conclusion, we discovered ODZ10117 as a novel STAT3-specific inhibitor for STAT3-targeted cancer therapy and demonstrated its anticancer activity in breast cancer models. ODZ10117 was identified by two-track screening assays using structure-based computational database screening and cell-based high-throughput screening. ODZ10117 well fits into the phospho-tyrosine binding pocket on the SH2 domain of STAT3 and the binding affinity of ODZ10117 to the SH2 domain of STAT3 was higher than the known STAT3 inhibitors such as S3I-201 and STA-21. ODZ10117 exhibited effective anticancer activities, which markedly suppressed tumor growth and metastasis of cancer cells and increased the survival rate in breast cancer xenograft models. However, some of the STAT3 inhibitors, such as Stattic [46] and WP1066 [47] showed more effective inhibition of STAT3 activation than ODZ10117 in the in vitro results that were analyzed from breast cancer cells, hepatocellular carcinoma cells, and glioma cells. Therefore, it is necessary to compare these molecules with ODZ10117. Based on these results, STAT3-targeted therapy should be developed for clinical trials.

## Supplementary Materials

The following are available online at https://www.mdpi.com/2077-0383/8/11/1847/s1, Figure S1: Molecular docking of ODZ17690 against the SH2 domain of STAT3. Figure S2: ODZ17690 is a hit compound of STAT3 inhibitor. Figure S3: Identification of ODZ10117 as a STAT3 inhibitor from the optimization of ODZ17690. Figure S4: 2D-interaction images of Pro704-pTyr705-Leu706 (A), S3I-201 (B), and STA-21 (C) are shown and various interaction types are color-coded. Figure S5: 1H NMR (A) and 13C NMR (B) spectra of the 2-(2,4-dichlorophenoxy)acetonitrile (A1). Figure S6: 1H NMR (A) and 13C NMR (B) spectra of the 2-(2,4-dichlorophenoxy)-N’-hydroxyacetimidamide (A2). Figure S7: 1H NMR (A) and 13C NMR (B) spectra of the ODZ10117. Figure S8: MDA-MB-231 cells were transfected with either HA- or Flag-tagged STAT3 plasmid, incubated for 24 h with either vehicle (0.1% DMSO) alone or ODZ10117 (40 μM), and then performed immunoblot analysis. Figure S9: ODZ10117 inhibits tyrosine phosphorylation of STAT3 in breast cancer. Figure S10: ODZ10117 does not affect other STAT family members and upstream regulators of STAT3. Figure S11: ODZ10117 does not affect body weight in the orthotopic metastasis model.

## References

[1] Levy D.E., Lee C.K. (2002). What does Stat3 do?. J. Clin. Investig..

[2] Schindler C., Plumlee C. (2008). Interferons pen the JAK-STAT pathway. Semin. Cell Dev. Biol..

[3] Akira S., Nishio Y., Inoue M., Wang X.J., Wei S., Matsusaka T., Yoshida K., Sudo T., Naruto M., Kishimoto T. (1994). Molecular cloning of APRF, a novel IFN-stimulated gene factor 3 p91-related transcription factor involved in the gp130-mediated signaling pathway. Cell.

[4] Zhong Z., Wen Z., Darnell J.E.J. (1994). Stat3: a STAT family member activated by tyrosine phosphorylation in response to epidermal growth factor and interleukin-6. Science.

[5] Huynh J., Chand A., Gough D., Ernst M. (2019). Therapeutically exploiting STAT3 activity in cancer—using tissue repair as a road map. Nat. Rev. Cancer.

[6] Kim B.H., Yi E.H., Ye S.K. (2016). Signal transducer and activator of transcription 3 as a therapeutic target for cancer and the tumor microenvironment. Arch. Pharm. Res..

[7] O’Shea J.J., Holland S.M., Staudt L.M. (2013). JAKs and STATs in immunity, immunodeficiency, and cancer. N. Engl. J. Med..

[8] Yu H., Lee H., Herrmann A., Buettner R., Jove R. (2014). Revisiting STAT3 signalling in cancer: new and unexpected biological functions. Nat. Rev. Cancer.

[9] Siegel R.L., Miller K.D., Jemal A. (2019). Cancer statistics, 2019. CA. Cancer J. Clin..

[10] Sørlie T., Perou C.M., Tibshirani R., Aas T., Geisler S., Johnsen H., Hastie T., Eisen M.B., van de Rijn M., Jeffrey S.S. (2001). Gene expression patterns of breast carcinomas distinguish tumor subclasses with clinical implications. Proc. Natl. Acad. Sci. USA.

[11] Sotiriou C., Neo S.Y., McShane L.M., Korn E.L., Long P.M., Jazaeri A., Martiat P., Fox S.B., Harris A.L., Liu E.T. (2003). Breast cancer classification and prognosis based on gene expression profiles from a population-based study. Proc. Natl. Acad. Sci. USA.

[12] Bianchini G., Balko J.M., Mayer I.A., Sanders M.E., Gianni L. (2016). Triple-negative breast cancer: challenges and opportunities of a heterogeneous disease. Nat. Rev. Clin. Oncol..

[13] Dai X., Xiang L., Li T., Bai Z. (2016). Cancer Hallmarks, Biomarkers and Breast Cancer Molecular Subtypes. J. Cancer.

[14] Cejalvo J.M., Pascual T., Fernández-Martínez A., Brasó-Maristany F., Gomis R.R., Perou C.M., Muñoz M., Prat A. (2018). Clinical implications of the non-luminal intrinsic subtypes in hormone receptor-positive breast cancer. Cancer Treat. Rev..

[15] Banerjee K., Resat H. (2016). Constitutive activation of STAT3 in breast cancer cells: A review. Int. J. Cancer.

[16] Sirkisoon S.R., Carpenter R.L., Rimkus T., Anderson A., Harrison A., Lange A.M., Jin G., Watabe K., Lo H.W. (2018). Interaction between STAT3 and GLI1/tGLI1 oncogenic transcription factors promotes the aggressiveness of triple-negative breast cancers and HER2-enriched breast cancer. Oncogene.

[17] Qin J.J., Yan L., Zhang J., Zhang W.D. (2019). STAT3 as a potential therapeutic target in triple negative breast cancer: a systematic review. J. Exp. Clin. Cancer Res..

[18] Kim B.H., Yi E.H., Li Y.C., Park I.C., Park J.Y., Ye S.K. (2019). Anticancer Activity of Tubulosine through Suppression of Interleukin-6-Induced Janus Kinase 2/Signal Transducer and Activation of Transcription 3 Signaling. J. Breast Cancer.

[19] Becker S., Groner B., Müller C.W. (1998). Three-dimensional structure of the Stat3beta homodimer bound to DNA. Nature.

[20] Sastry G.M., Adzhigirey M., Day T., Annabhimoju R., Sherman W. (2013). Protein and ligand preparation: parameters, protocols, and influence on virtual screening enrichments. J. Comput. Aided Mol. Des..

[21] Chen I.J., Foloppe N. (2010). Drug-like bioactive structures and conformational coverage with the LigPrep/ConfGen suite: comparison to programs MOE and catalyst. J. Chem. Inf. Model..

[22] Halgren T.A., Murphy R.B., Friesner R.A., Beard H.S., Frye L.L., Pollard W.T., Banks J.L. (2004). Glide: A new approach for rapid, accurate docking and scoring. 2. Enrichment factors in database screening. J. Med. Chem..

[23] Siddiquee K., Zhang S., Guida W.C., Blaskovich M.A., Greedy B., Lawrence H.R., Yip M.L., Jove R., McLaughlin M.M., Lawrence N.J. (2007). Selective chemical probe inhibitor of Stat3, identified through structure-based virtual screening, induces antitumor activity. Proc. Natl. Acad. Sci. USA.

[24] Song H., Wang R., Wang S., Lin J. (2005). A low-molecular-weight compound discovered through virtual database screening inhibits Stat3 function in breast cancer cells. Proc. Natl. Acad. Sci. USA.

[25] Shin D.S., Kim H.N., Shin K.D., Yoon Y.J., Kim S.J., Han D.C., Kwon B.M. (2009). Cryptotanshinone inhibits constitutive signal transducer and activator of transcription 3 function through blocking the dimerization in DU145 prostate cancer cells. Cancer Res..

[26] Kim B.H., Yin C.H., Guo Q., Bach E.A., Lee H., Sandoval C., Jayabose S., Ulaczyk-Lesanko A., Hall D.G., Baeg G.H. (2008). A small-molecule compound identified through a cell-based screening inhibits JAK/STAT pathway signaling in human cancer cells. Mol. Cancer Ther..

[27] Nelson E.A., Walker S.R., Kepich A., Gashin L.B., Hideshima T., Ikeda H., Chauhan D., Anderson K.C., Frank D.A. (2008). Nifuroxazide inhibits survival of multiple myeloma cells by directly inhibiting STAT3. Blood.

[28] Li Y., Rogoff H.A., Keates S., Gao Y., Murikipudi S., Mikule K., Leggett D., Li W., Pardee A.B., Li C.J. (2015). Suppression of cancer relapse and metastasis by inhibiting cancer stemness. Proc. Natl. Acad. Sci. USA..

[29] Renga J.M., Wang P.C. (1984). The Salt-Free Synthesis of Aryl Ethers Using Methyl Trichloracetate. Synth. Commun..

[30] Eloy F., Lenaers R. (1962). The Chemistry of Amidoximes and Related Compounds. Chem. Rev..

[31] Augustine J.K., Akabote V., Hegde S.G., Alagarsamy P. (2009). PTSA-ZnCl2: an efficient catalyst for the synthesis of 1, 2, 4-oxadiazoles from amidoximes and organic nitriles. J. Org. Chem..

[32] Johnson D.E., O’Keefe R.A., Grandis J.R. (2018). Targeting the IL-6/JAK/STAT3 signalling axis in cancer. Nat. Rev. Clin. Oncol..

[33] Miklossy G., Hilliard T.S., Turkson J. (2013). Therapeutic modulators of STAT signalling for human diseases. Nat. Rev. Drug Discov..

[34] Clark A.G., Vignjevic D.M. (2015). Modes of cancer cell invasion and the role of the microenvironment. Curr. Opin. Cell Biol..

[35] Hynes N.E., Gullick W. (2006). Therapeutic targeting of signal transduction pathways and proteins in breast cancer. J. Mammary Gland Biol. Neoplasia.

[36] Azab S., Al-Hendy A. (2015). Signal Transduction Pathways in Breast Cancer—Drug Targets and Challenges. Breast Cancer—Carcinogenesis, Cell Growth and Signalling Pathways.

[37] Chai E.Z., Shanmugam M.K., Arfuso F., Dharmarajan A., Wang C., Kumar A.P., Samy R.P., Lim L.H., Wang L., Goh B.C. (2016). Targeting transcription factor STAT3 for cancer prevention and therapy. Pharmacol. Ther..

[38] Huynh J., Etemadi N., Hollande F., Ernst M., Buchert M. (2017). The JAK/STAT3 axis: A comprehensive drug target for solid malignancies. Semin. Cancer Biol..

[39] Spitzner M., Ebner R., Wolff H.A., Ghadimi B.M., Wienands J., Grade M. (2014). STAT3: A Novel Molecular Mediator of Resistance to Chemoradiotherapy. Cancers (Basel).

[40] Baudino T.A. (2015). Targeted Cancer Therapy: The Next Generation of Cancer Treatment. Curr. Drug Discov. Technol..

[41] Brown C. (2016). Targeted therapy: An elusive cancer target. Nature.

[42] Banerjee S., Biehl A., Gadina M., Hasni S., Schwartz D.M. (2017). JAK-STAT Signaling as a Target for Inflammatory and Autoimmune Diseases: Current and Future Prospects. Drugs.

[43] Dimri S., Sukanya S., De A. (2017). Approaching non-canonical STAT3 signaling to redefine cancer therapeutic strategy. Integr. Mol. Med..

[44] Furtek S.L., Backos D.S., Matheson C.J., Reigan P. (2016). Strategies and Approaches of Targeting STAT3 for Cancer Treatment. ACS Chem. Biol..

[45] Wong A.L.A., Hirpara J.L., Pervaiz S., Eu J.Q., Sethi G., Goh B.C. (2017). Do STAT3 inhibitors have potential in the future for cancer therapy?. Expert Opin. Investig. Drugs.

[46] Schust J., Sperl B., Hollis A., Mayer T.U., Berg T. (2006). Stattic: A small-molecule inhibitor of STAT3 activation and dimerization. Chem. Biol..

[47] Iwamaru A., Szymanski S., Iwado E., Aoki H., Yokoyama T., Fokt I., Hess K., Conrad C., Madden T., Sawaya R. (2007). A novel inhibitor of the STAT3 pathway induces apoptosis in malignant glioma cells both in vitro and in vivo. Oncogene.

