# Supplementary material for: Development of Oxadiazole-Based ODZ10117 as a Small-Molecule Inhibitor of STAT3 for Targeted Cancer Therapy
Source: J Clin Med. 2019 Nov 2;8(11):1847. doi: 10.3390/jcm8111847 (PMC6912340; doi:10.3390/jcm8111847)
Supplement: Supplementary file 1 [file jcm-08-01847-s001.zip › jcm-592777R_supplementary materials figure.pptx]

## Slide 1
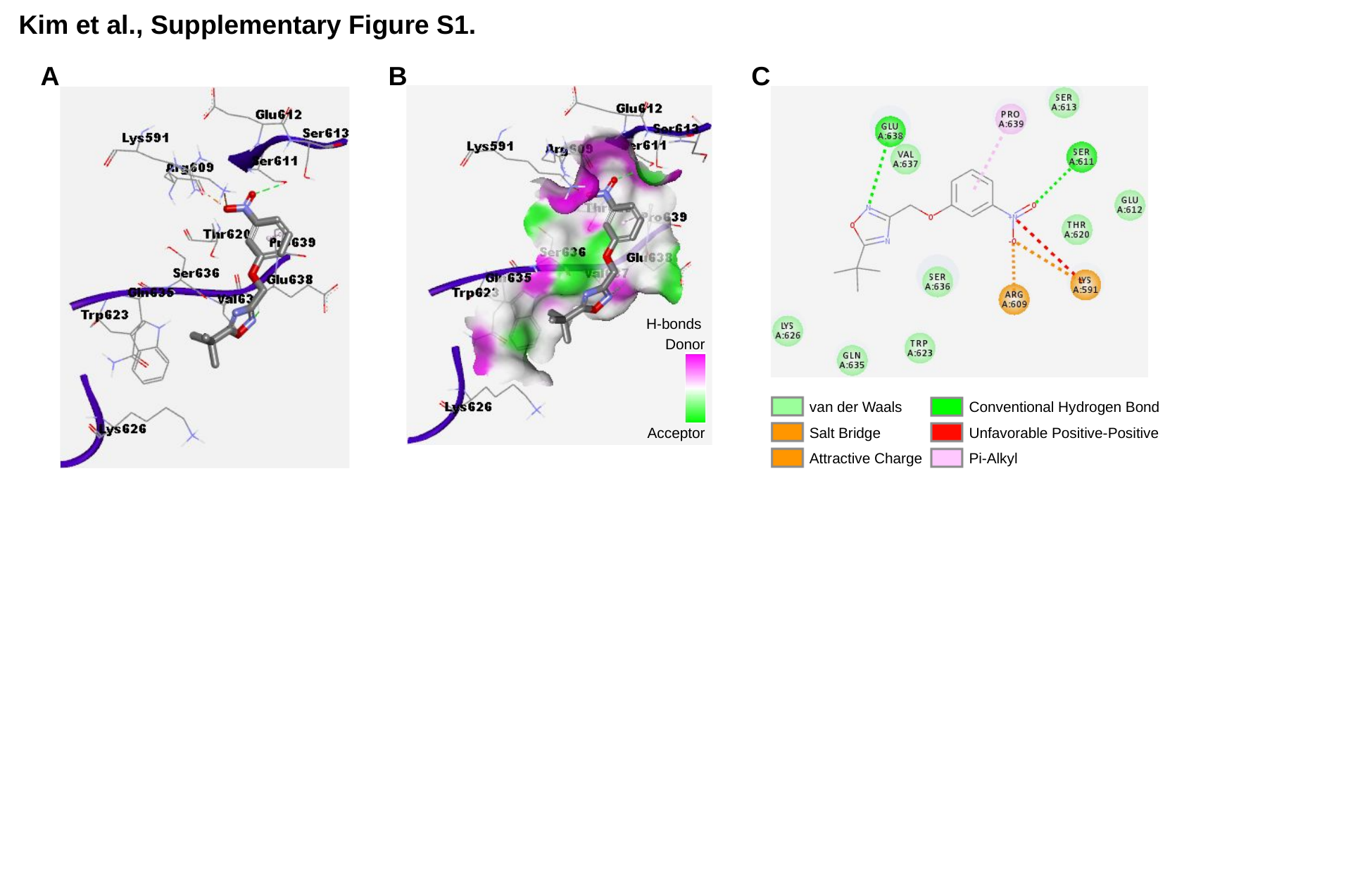

Kim et al., Supplementary Figure S1.
A
B
C
Donor
Acceptor
van der Waals
Conventional Hydrogen Bond
Salt Bridge
Unfavorable Positive-Positive
Attractive Charge
Pi-Alkyl
H-bonds

## Slide 2
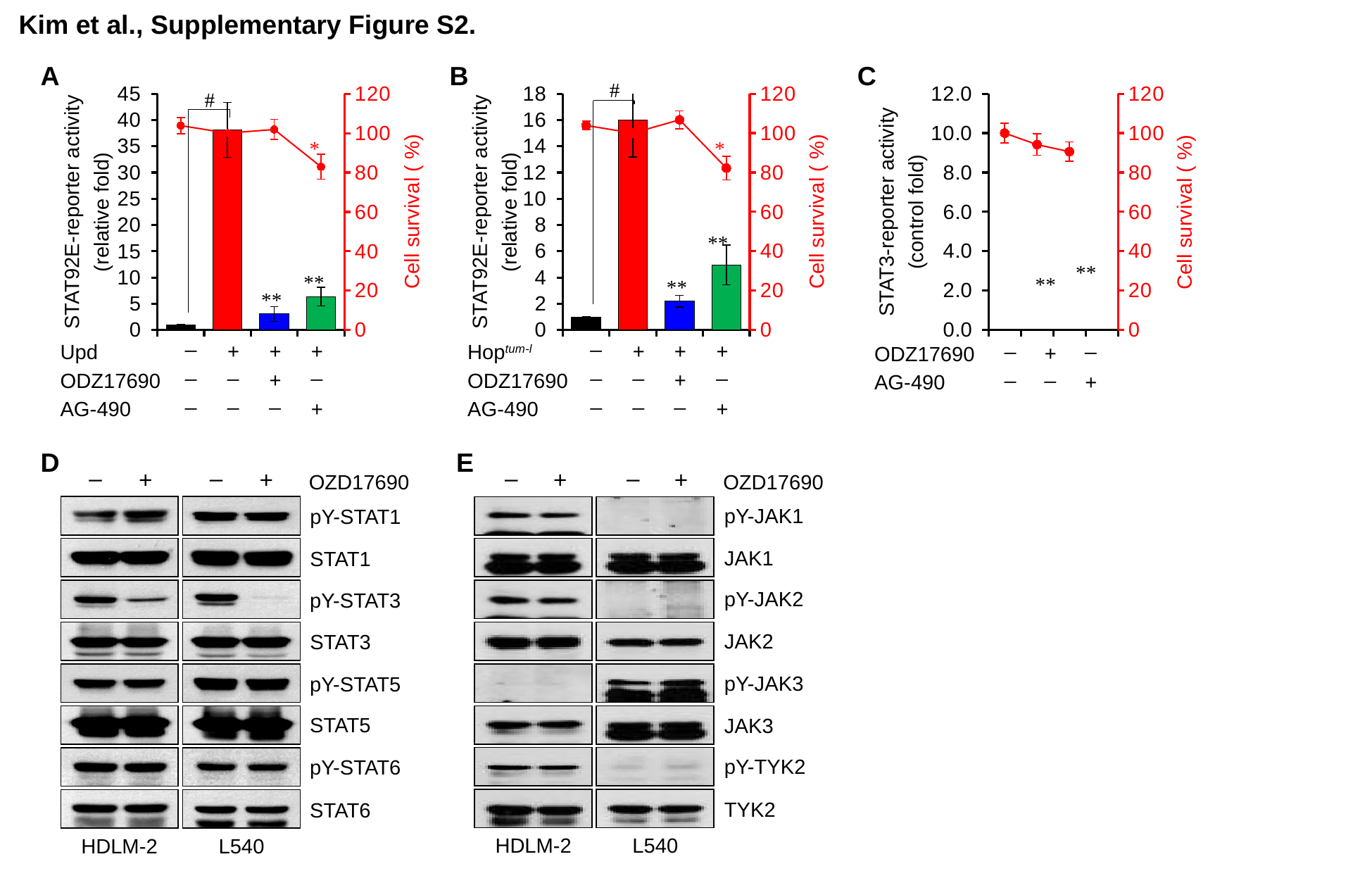

Kim et al., Supplementary Figure S2.
A
B
C
#
### Chart
| Category | | |
|---|---|---|
| Vehicle | 1.0 | 103.88315190151727 |
| Vehicle | 15.97615261158145 | 100.0 |
| 17690 | 2.1946798475150184 | 106.74954191404159 |
| AG | 4.962220629131467 | 82.23640766404029 |*
STAT92E-reporter activity
(relative fold)
Cell survival ( %)
**
**
_
+
+
+
Hoptum-l
_
_
_
+
ODZ17690
_
_
_
+
AG-490
### Chart
| Category | | |
|---|---|---|
| Vehicle | 1.0 | 103.83459681731051 |
| Vehicle | 38.13020210559966 | 100.0 |
| 17690 | 3.0391515050497424 | 101.84896980441803 |
| AG | 6.343812586413968 | 82.90442307338512 |#
*
STAT92E-reporter activity
(relative fold)
Cell survival ( %)
**
**
_
+
+
+
Upd
_
_
_
+
ODZ17690
_
_
_
+
AG-490
### Chart
| Category | | |
|---|---|---|
| Vehicle | 1.0 | 100.0 |
| 17690 | 0.1688806971876855 | 94.1474106850213 |
| AG | 0.21291122657584058 | 90.58044493608654 |STAT3-reporter activity
(control fold)
Cell survival ( %)
**
**
_
_
+
ODZ17690
_
_
+
AG-490
D
E
–
–
+
+
OZD17690
pY-STAT1
STAT1
pY-STAT3
STAT3
pY-STAT5
STAT5
pY-STAT6
STAT6
HDLM-2
L540
–
–
+
+
OZD17690
pY-JAK1
JAK1
pY-JAK2
JAK2
pY-JAK3
JAK3
pY-TYK2
TYK2
HDLM-2
L540

## Slide 3
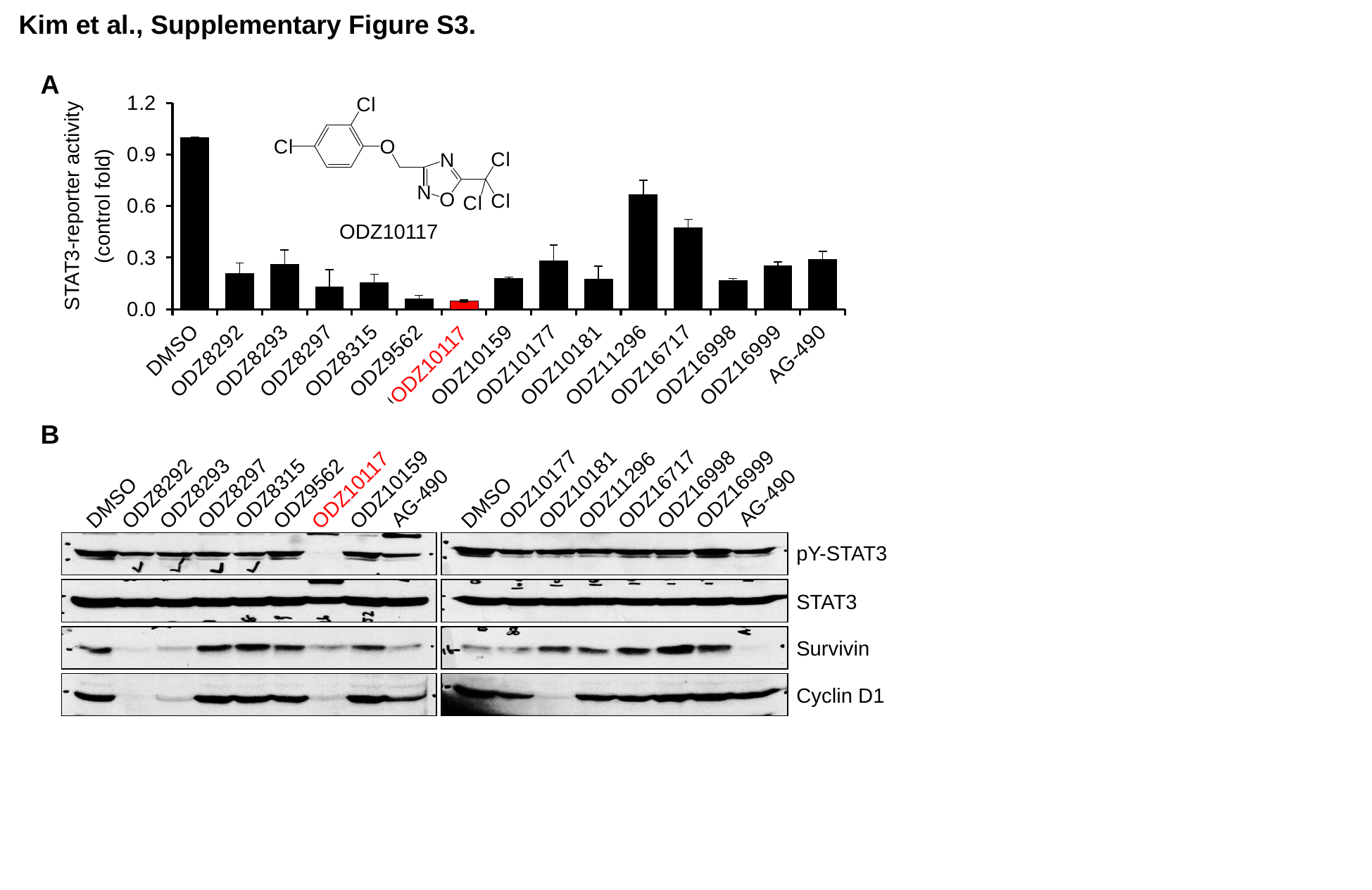

Kim et al., Supplementary Figure S3.
A
### Chart
| Category | |
|---|---|
| DMSO | 1.0 |
| ODZ8292 | 0.20785593265336988 |
| ODZ8293 | 0.2589954511197299 |
| ODZ8297 | 0.1315849607012652 |
| ODZ8315 | 0.1554280512215765 |
| ODZ9562 | 0.060128338173807624 |
| ODZ10117 | 0.047277177833429204 |
| ODZ10159 | 0.17855009340482908 |
| ODZ10177 | 0.2792506674524301 |
| ODZ10181 | 0.1755547535243308 |
| ODZ11296 | 0.6643807537814039 |
| ODZ16717 | 0.47577392889127545 |
| ODZ16998 | 0.16792156108216888 |
| ODZ16999 | 0.25402502463619697 |
| AG-490 | 0.2897661924625581 |ODZ10117
STAT3-reporter activity (control fold)
ODZ10117
B
ODZ10117
ODZ10159
ODZ10177
ODZ10181
ODZ11296
ODZ16717
ODZ16998
ODZ16999
ODZ8292
ODZ8293
ODZ8297
ODZ8315
ODZ9562
AG-490
AG-490
DMSO
DMSO
pY-STAT3
STAT3
Survivin
Cyclin D1

## Slide 4
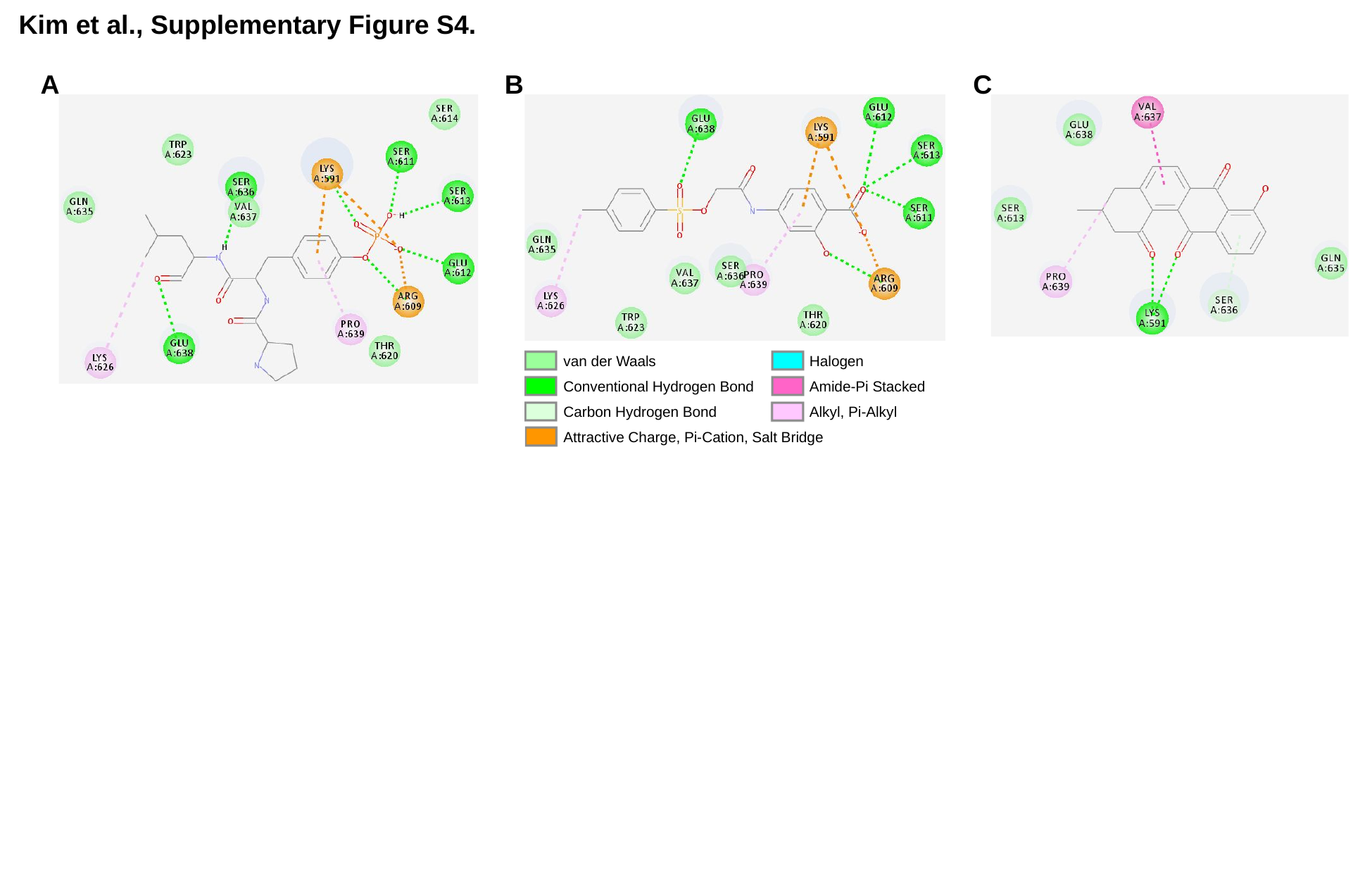

Kim et al., Supplementary Figure S4.
A
B
C
van der Waals
Halogen
Conventional Hydrogen Bond
Amide-Pi Stacked
Alkyl, Pi-Alkyl
Carbon Hydrogen Bond
Attractive Charge, Pi-Cation, Salt Bridge

## Slide 5
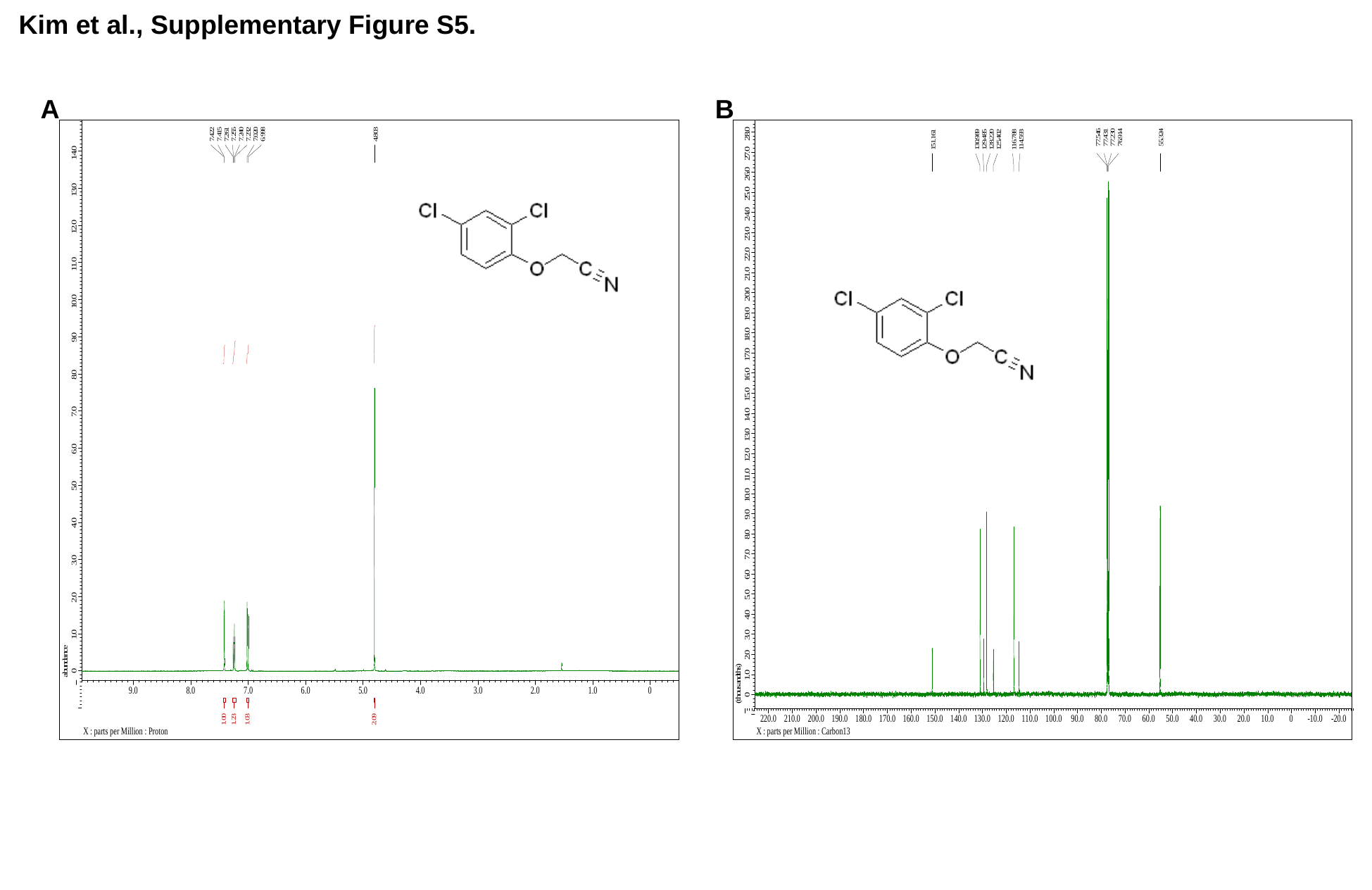

Kim et al., Supplementary Figure S5.
A
B

## Slide 6
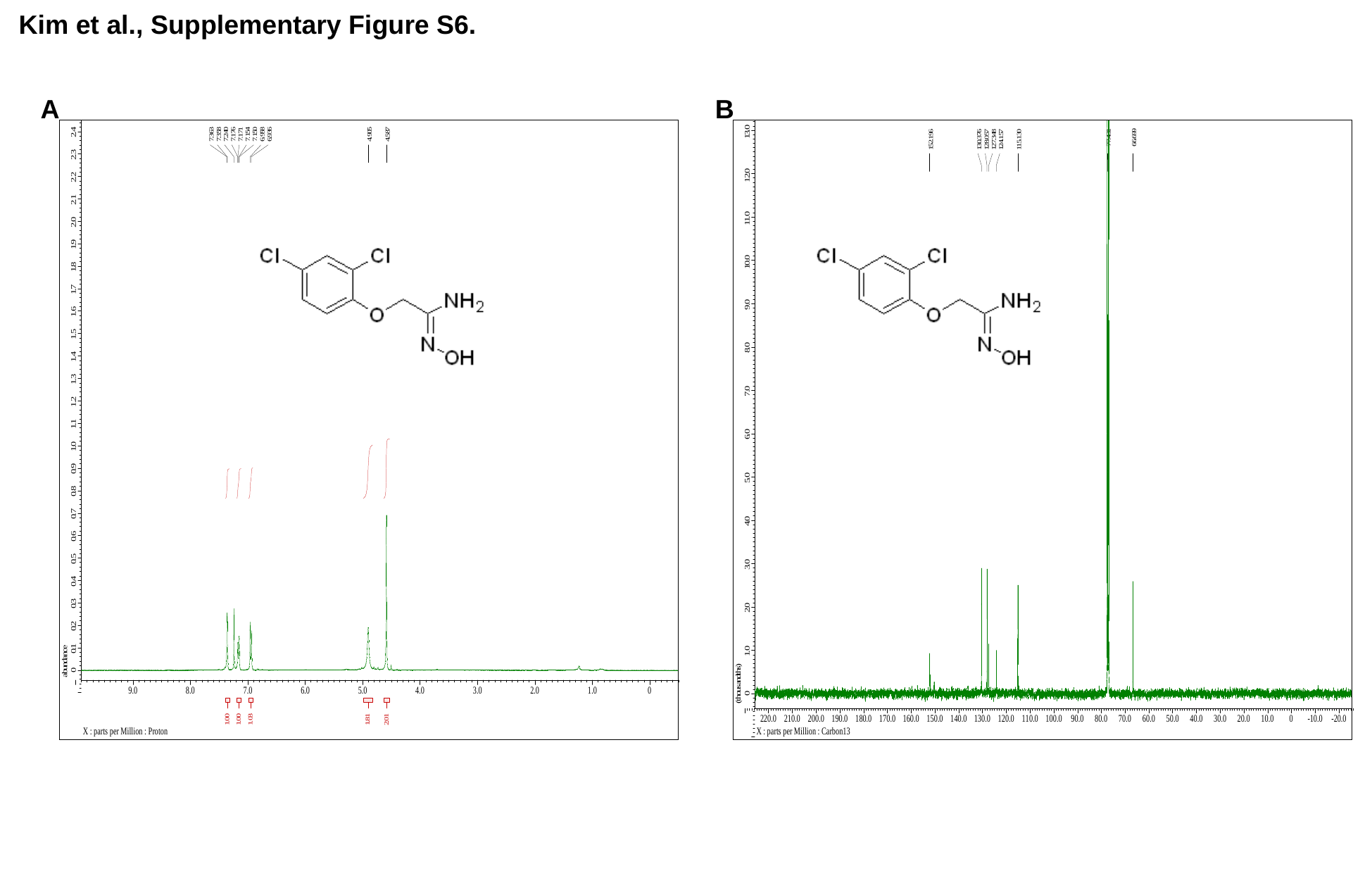

Kim et al., Supplementary Figure S6.
A
B

## Slide 7
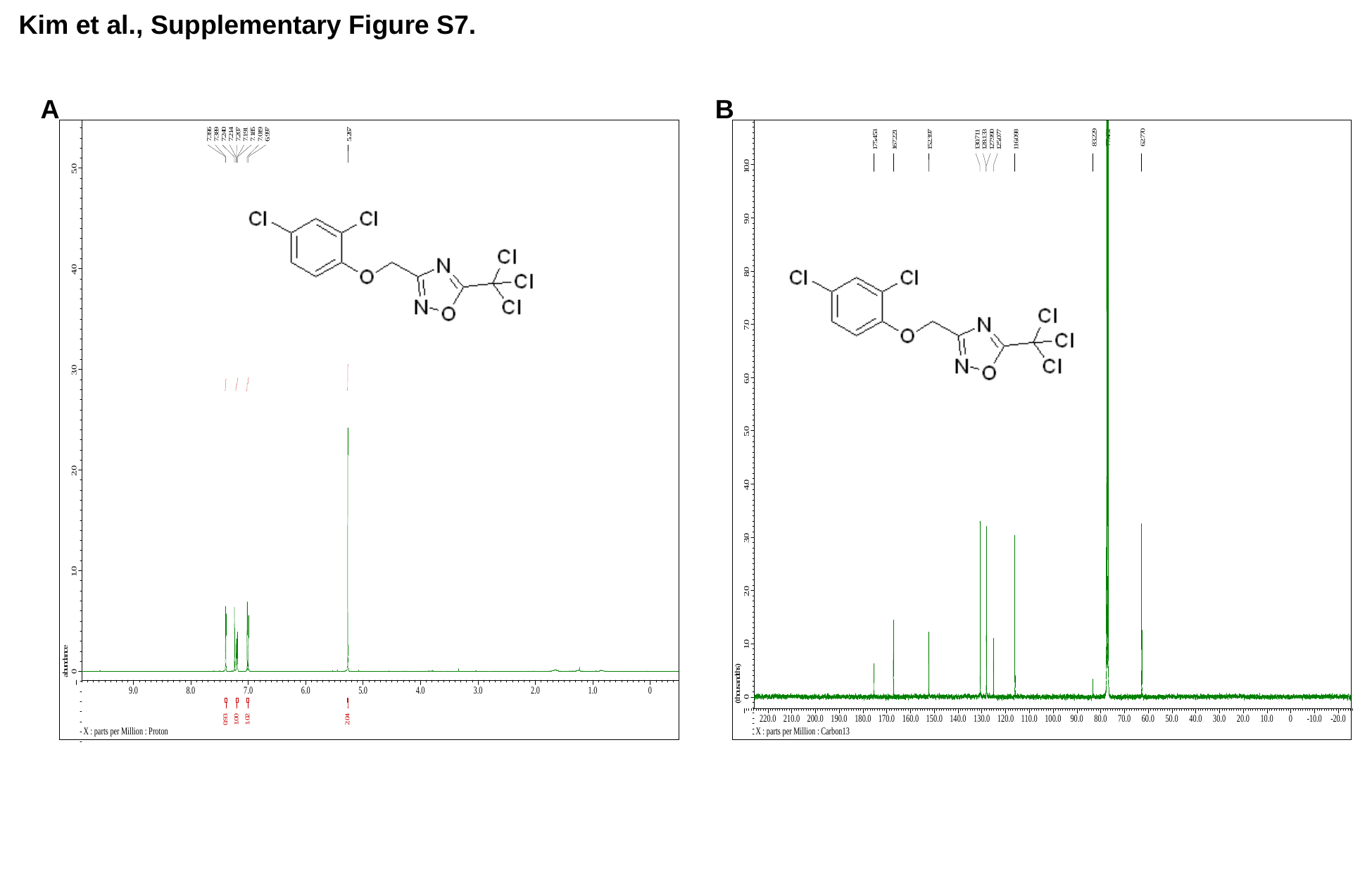

Kim et al., Supplementary Figure S7.
A
B

## Slide 8
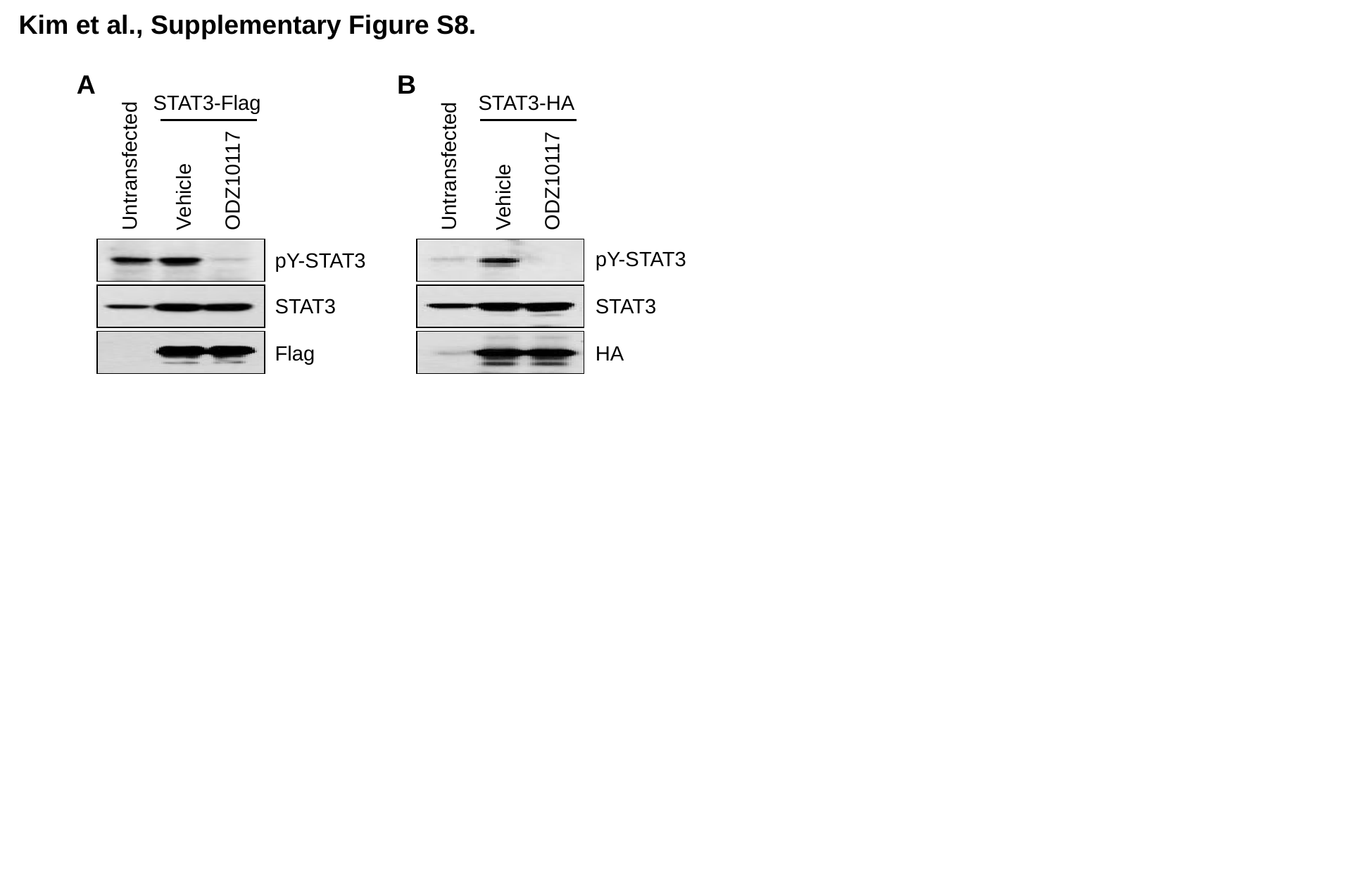

Kim et al., Supplementary Figure S8.
A
B
STAT3-Flag
Untransfected
Vehicle
ODZ10117
pY-STAT3
STAT3
Flag
STAT3-HA
Untransfected
Vehicle
ODZ10117
pY-STAT3
STAT3
HA

## Slide 9
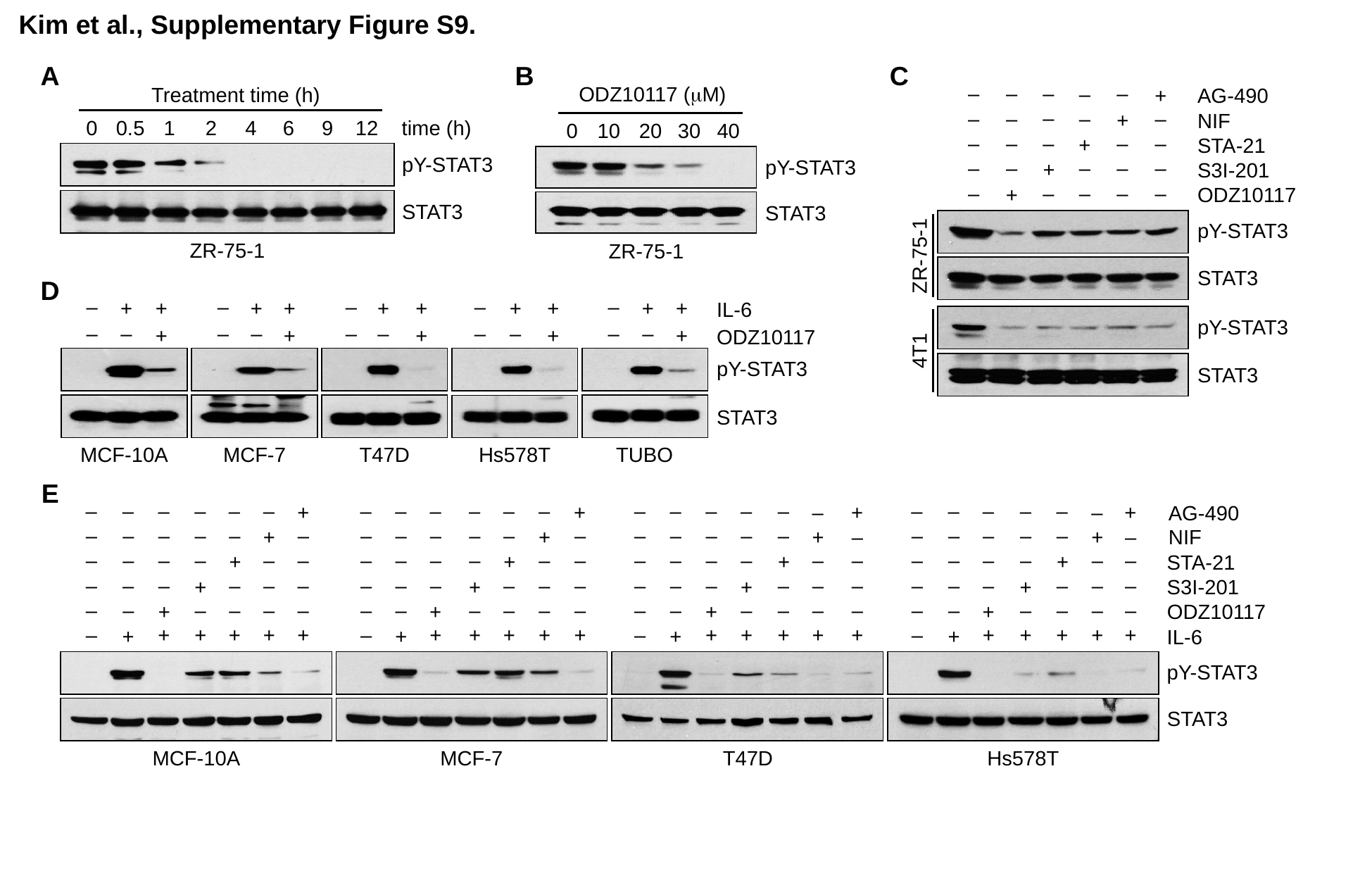

Kim et al., Supplementary Figure S9.
A
B
C
–
–
–
–
–
+
AG-490
–
–
–
–
–
+
NIF
–
–
–
–
–
+
STA-21
–
–
–
–
–
+
S3I-201
–
–
–
–
–
+
ODZ10117
ZR-75-1
pY-STAT3
STAT3
4T1
pY-STAT3
STAT3
ODZ10117 (μM)
0
10
20
30
40
pY-STAT3
STAT3
ZR-75-1
Treatment time (h)
0
0.5
1
2
4
6
9
12
time (h)
pY-STAT3
STAT3
ZR-75-1
D
–
–
–
–
–
+
+
+
+
+
+
+
+
+
+
IL-6
–
–
–
–
–
–
–
–
–
–
+
+
+
+
+
ODZ10117
pY-STAT3
STAT3
MCF-10A
MCF-7
T47D
Hs578T
TUBO
E
–
–
–
–
–
–
–
–
+
–
–
–
–
+
–
–
–
–
–
–
+
–
–
–
–
–
+
–
–
–
–
–
+
+
+
+
+
+
+
–
–
–
–
–
–
+
–
–
–
–
–
+
–
–
–
–
–
–
–
+
–
–
–
–
–
–
+
–
–
–
–
–
–
+
–
+
+
+
+
+
+
–
–
–
–
–
–
–
–
–
–
+
+
AG-490
–
–
–
–
–
–
–
–
–
–
–
–
–
+
+
NIF
–
–
–
–
–
–
–
–
–
–
–
–
–
+
+
STA-21
–
–
–
–
–
–
–
–
–
–
–
–
–
+
+
S3I-201
–
–
–
–
–
–
–
–
–
–
–
–
–
+
+
ODZ10117
–
+
+
+
+
+
–
+
+
+
+
+
–
+
+
IL-6
pY-STAT3
STAT3
MCF-10A
MCF-7
T47D
Hs578T

## Slide 10
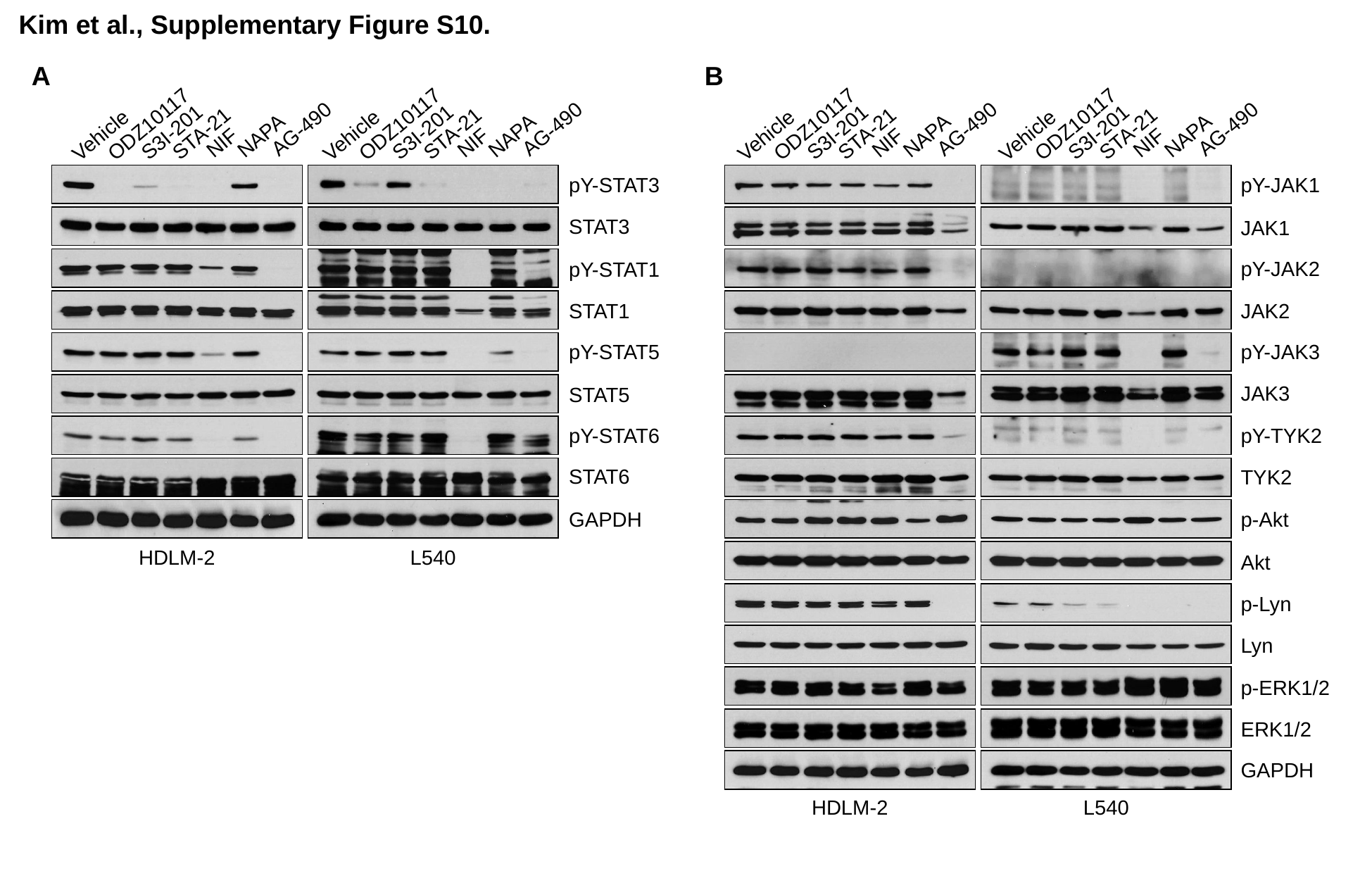

Kim et al., Supplementary Figure S10.
A
B
ODZ10117
ODZ10117
ODZ10117
ODZ10117
AG-490
AG-490
AG-490
AG-490
S3I-201
S3I-201
S3I-201
STA-21
NAPA
NAPA
NAPA
NAPA
STA-21
S3I-201
Vehicle
Vehicle
Vehicle
Vehicle
STA-21
STA-21
NIF
NIF
NIF
NIF
pY-STAT3
pY-JAK1
STAT3
JAK1
pY-JAK2
pY-STAT1
STAT1
JAK2
pY-STAT5
pY-JAK3
JAK3
STAT5
pY-STAT6
pY-TYK2
STAT6
TYK2
GAPDH
p-Akt
HDLM-2
L540
Akt
p-Lyn
Lyn
p-ERK1/2
ERK1/2
GAPDH
HDLM-2
L540

## Slide 11
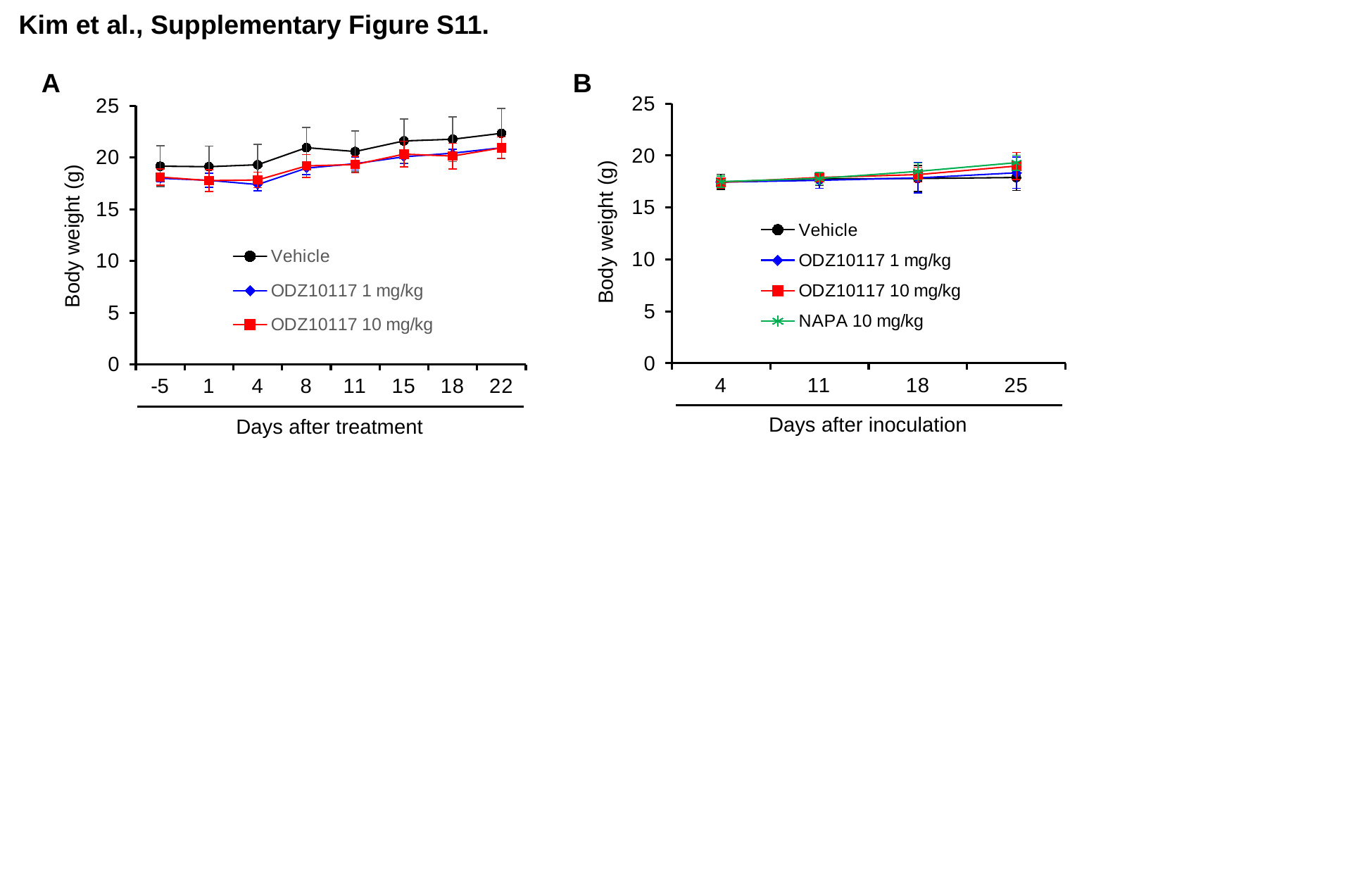

Kim et al., Supplementary Figure S11.
### Chart
| Category | Vehicle | ODZ10117 1 mg/kg | ODZ10117 10 mg/kg |
|---|---|---|---|
| -5 | 19.169999999999998 | 18.006 | 18.12 |
| 1 | 19.124000000000002 | 17.802 | 17.778 |
| 4 | 19.312 | 17.396 | 17.842 |
| 8 | 20.964 | 18.984 | 19.198 |
| 11 | 20.592 | 19.418 | 19.332 |
| 15 | 21.618000000000002 | 20.08 | 20.337999999999997 |
| 18 | 21.778 | 20.433999999999997 | 20.152 |
| 22 | 22.354000000000006 | 20.952 | 20.946 |Body weight (g)
Days after treatment
A
B
### Chart
| Category | Vehicle | ODZ10117 1 mg/kg | ODZ10117 10 mg/kg | NAPA 10 mg/kg |
|---|---|---|---|---|
| 4 | 17.466 | 17.440999999999995 | 17.422999999999995 | 17.488999999999997 |
| 11 | 17.750000000000004 | 17.614 | 17.892000000000003 | 17.799 |
| 18 | 17.793000000000003 | 17.848999999999997 | 18.166666666666668 | 18.477999999999998 |
| 25 | 17.887000000000004 | 18.34 | 18.993333333333332 | 19.31833333333333 |Body weight (g)
Days after inoculation
