# Supplementary material for: Development of Oxadiazole-Based ODZ10117 as a Small-Molecule Inhibitor of STAT3 for Targeted Cancer Therapy
Source: J Clin Med. 2019 Nov 2;8(11):1847. doi: 10.3390/jcm8111847 (PMC6912340; doi:10.3390/jcm8111847)
Supplement: Supplementary file 1 [file jcm-08-01847-s001.zip › jcm-592777R_supplementary materials.docx]

**Development of Oxadiazole-based ODZ10117 as a Small-Molecule Inhibitor of STAT3 for Targeted Cancer Therapy**

Byung-Hak Kim^1,2,3,†^, Haeri Lee^1,2,†^, Yeonghun Song^4^, Joon-Suk Park^5^, Changdev G. Gadhe^6^, Jiwon Choi^1^, Chung-Gi Lee^3^, Ae Nim Pae^6,7^, Sanghee Kim^4^, and Sang-Kyu Ye^1,2,8,9,^*

^1^Department of Pharmacology and Biomedical Sciences, and ^2^Biomedical Science Project (BK21^PLUS^), Seoul National University College of Medicine, Seoul, Republic of Korea

^3^CYTUS H&B Corporation, Cheongju, Republic of Korea

^4^College of Pharmacy, Seoul National University, Seoul, Republic of Korea

^5^Laboratory Animal Center, Daegu-Gyeongbuk Medical Innovation Foundation, Daegu, Republic of Korea

^6^Convergence Research Center for Diagnosis, Treatment and Care System of Dementia, Korea Institute of Science and Technology, Seoul, Republic of Korea

^7^Division of Bio-Medical Science &Technology, KIST School, Korea University of Science and Technology, Seoul, Republic of Korea

^8^Ischemic/Hypoxic Disease Institute, Seoul National University College of Medicine, Seoul, Republic of Korea

^9^Neuro-Immune Information Storage Network Research Center, Seoul National University College of Medicine, Seoul, Republic of Korea

^†^These authors contributed equally.

**^*^Corresponding author:** [sangkyu@snu.ac.kr](mailto:sangkyu@snu.ac.kr) (S.K.Y.)

**Table of Contents**

**1. Supplementary Results**

**2. Supplementary Figure Legends**

**1. Supplementary Results**

*Chemical synthesis*

*General information*: We used only reagent-grade chemicals purchased from Sigma-Aldrich. All of the reactions were performed under an inert atmosphere consisting of dry nitrogen and using distilled dry solvents. The reactions were monitored by thin layer chromatography (TLC) analysis using pre-coated Merck silica gel Kiesegel 60 F254 TLC plates. The compound spots were visualized using UV light (254 nm) and by staining with either potassium permanganate or anisaldehyde solutions. Flash column chromatography was performed on silica gel (230-400 mesh). The melting points were measured using a Buchi B-540 melting point apparatus without correction. ^1^H NMR (400, 600, or 800 MHz) and ^13^C NMR (100, 150, or 200 MHz) spectra were recorded in δ units relative to the non-deuterated solvent used as the internal reference (CDCl_3_‒*d_1_*: 7.24 ppm/^1^H NMR, 77.0 ppm/^13^C NMR). The infrared (IR) spectra were recorded on a Fourier transform infrared spectrometer. High-resolution mass spectra (HRMS) were recorded using fast atom bombardment (FAB).

*Synthesis of 2-(2,4-dichlorophenoxy)acetonitrile (A1)*: K_2_CO_3_ (424 mg, 3.07 mmol) was added to a solution of 2,4-dichlorophenol (500 mg, 3.07 mmol) in dimethylformamide (DMF, 4.4 mL) at room temperature. The reaction mixture was added to bromoacetonitrile (97%, 0.220 mL, 3.07 mmol) in DMF (1.3 mL) and stirred at room temperature for 5 h. The reaction mixture was quenched by the addition of H_2_O, and then extracted with ethyl acetate (EtOAc) three times. The combined organic layers were dried over magnesium sulfate (MgSO_4_) and concentrated *in vacuo*. The residue was purified by flash chromatography on silica gel (hexane/EtOAc, 3:1) to yield 2-(2,4-dichlorophenoxy)acetonitrile (A2, 607 mg, 98%) as a white solid with melting point 50.7–52.0°C; ^1^H NMR (400 MHz, CDCl_3_) δ 7.42 (d, *J* = 2.8 Hz, 1H), 7.25 (dd, *J* = 9.0, 2.6 Hz, 1H), 7.01 (d, *J* = 8.8 Hz, 1H), 4.80 (s, 2H); ^13^C NMR (100 MHz, CDCl_3_) δ 151.2, 131.0, 129.5, 128.2, 125.4, 116.8, 114.6, 55.3; IR (near, cm^–1^) υ_max_ 1478, 1444, 1292, 1265, 1230, 1105, 1069, 1034, 802, and 712.

*Synthesis of 2-(2,4-dichlorophenoxy)-N'-hydroxyacetimidamide (A2)*: Hydroxylamine hydrochloride (NH_2_OH∙HCl, 235 mg, 3.38 mmol) was added to a solution of trimethylamine (Et_3_N, 0.471 mL, 3.38 mmol) in ethanol/H_2_O (1:1, 3.6 mL) at room temperature and stirred for 5 min. The reaction mixture was added to a solution of A2 (521 mg, 2.58 mmol) in ethanol (14.3 mL) and stirred at 100°C for 1 h. Then, ice water was added to the mixture and the suspension was filtered. The filter cake was washed with water and dried *in vacuo* to 2-(2,4-dichlorophenoxy)-*N'*-hydroxyacetimidamide (A3, 514.7 mg, 85%) as a white solid with melting point 99.5–103°C; ^1^H NMR (400 MHz, CDCl_3_) δ 7.36 (d, *J* = 2.0 Hz, 1H), 7.16 (dd, *J* = 8.8, 2.0 Hz, 1H), 6.95 (d, *J* = 8.80 Hz, 1H), 4.91 (brs, 2H), 4.59 (s, 2H); ^13^C NMR (100 MHz, CDCl_3_) δ 152.2, 130.4, 128.1, 127.3, 124.2, 115.1 (2C), 66.7; IR (near, cm^–1^) υ_max_ 3485, 3102, 2813, 1671, 1479, 1288, 1264, 1228, 1104, 1063, 802, 765; HRMS (FAB): calculated for C_8_H_9_Cl_2_N_2_O_2_ [M+H]^+^ 235.0041, found 235.0040.

*Synthesis of ODZ10117*: To a solution of A3 (100 mg, 0.43 mmol) in DMF (1.0 mL), trichloroacetonitrile (0.040 mL, 0.43 mmol), *p*-toluenesulfonic acid monohydrate (41 mg, 0.22 mmol), and zinc chloride (30 mg, 0.22 mmol) were added. The reaction mixture was stirred at 80°C for 16 h, then cooled to room temperature and quenched by saturated NaHCO_3_ solution. The resulting mixture was extracted with EtOAc three times and the combined organic layers were dried over MgSO4, filtered, and concentrated *in vacuo*. The crude residue was purified by flash chromatography on silica gel (hexane:EtOAc, 10:1) to yield ODZ10117 (A4, 81 mg, 53%) as a colorless oil with ^1^H NMR (400 MHz, CDCl_3_) δ 7.39 (d, *J* = 2.8 Hz, 1H), 7.20 (dd, *J* = 9.0, 2.6 Hz, 1H), 7.01 (d, *J* = 8.80 Hz, 1H), 5.27 (s, 2H); ^13^C NMR (100 MHz, CDCl_3_) δ 175.5, 167.2, 152.4, 130.7, 128.1, 128.0, 125.1, 116.1, 83.2, 62.8; IR (near, cm^–1^) υ_max_ 3452, 2930, 1621, 1575, 1480, 1289, 1262, 1230, 1105, 1069, 852, 822, 802, 720; HRMS (FAB): calculated for C_10_H_6_Cl_5_N_2_O_2_ [M+H]^+^ 360.8872, found 360.8872.

**2. Supplementary Figure Legends**

**Supplementary Figure S1.** Molecular docking of ODZ17690 against the SH2 domain of STAT3. (A) Docked model of ODZ17690 is shown in stick and the surrounding residues of the SH2 domain of STAT3 are shown by a line model. Hydrogen bonds are shown for Ser611 and Glu638. A salt bridge and attractive charge type interactions are observed between the Lys591 and Arg609 and nitro group O of ODZ17690. (B) Transparent hydrogen bond acceptor/donor surface is shown for surrounding residues. (C) 2D-interaction image is shown and various interaction types are color coded.

**Supplementary Figure S2.** ODZ17690 is a hit compound of STAT3 inhibitor. (A and B) Cytokine (Upd)- or Hop*^tum-l^* (a dominant mutation of JAK)-induced S2-NP/STAT92E-Luc cells were incubated for 24 h with vehicle (0.1% DMSO) alone, ODZ17690 (150 μM), or AG-490 (10 μM), and STAT92E-reporter activity was determined. Results are represented as mean ± SEM of three independent experiments (*n* = 3). ^#^*P* < 0.005 compared to the vehicle-treated group and ^**^*P* < 0.005 compared to the Upd- or Hop*^tum-l^*-induced group. (C) MDA-MB-231/STAT3-Luc cells were incubated for 24 h with vehicle (0.1% DMSO) alone, ODZ17690 (150 μM), or AG-490 (150 μM), and STAT3-reporter activity and cell viability were determined. Results are represented as mean ± SEM of three independent experiments (*n* = 3). ^**^*P* < 0.005 compared to the vehicle-treated group. (D and E) Human Hodgkin's lymphoma HDLM-2 and L540 cells were incubated for 24 h with ODZ17690 (150 μM) and then performed immunoblot analysis.

**Supplementary Figure S3.** Identification of ODZ10117 as a STAT3 inhibitor from the optimization of ODZ17690. (A) MDA-MB-231/STAT3-Luc cells were incubated for 24 h with vehicle (0.1% DMSO) alone or each ODZ17690 derivative (40 μM), and STAT3-reporter activity was determined. Results are represented as mean ± SEM of three independent experiments (*n* = 3). Chemical structure of ODZ10117 is represented. (B) MDA-MB-231 cells were incubated for 24 h with either vehicle (0.1% DMSO) or each compound (40 μM) and immunoblot analysis was performed. Results shown represent only 13 compounds of the 144 derivatives of ODZ17690. AG-490 was used as a positive control.

**Supplementary Figure S4.** 2D-interaction images of Pro704-pTyr705-Leu706 (A), S3I-201 (B), and STA-21 (C) are shown and various interaction types are color-coded.

**Supplementary Figure S5.** ^1^H NMR (A) and ^13^C NMR (B) spectra of the 2-(2,4-dichlorophenoxy)acetonitrile (A1).

**Supplementary Figure S6.** ^1^H NMR (A) and ^13^C NMR (B) spectra of the 2-(2,4-dichlorophenoxy)-*N'*-hydroxyacetimidamide (A2).

**Supplementary Figure S7.** ^1^H NMR (A) and ^13^C NMR (B) spectra of the ODZ10117.

**Supplementary Figure S8.** MDA-MB-231 cells were transfected with either HA- or Flag-tagged STAT3 plasmid, incubated for 24 h with either vehicle (0.1% DMSO) alone or ODZ10117 (40 μM), and then performed immunoblot analysis.

**Supplementary Figure S9.** ODZ10117 inhibits tyrosine phosphorylation of STAT3 in breast cancer. (A and B) Cells were incubated with 40 μM ODZ10117 in a time-dependent manner (A) or incubated for 9 h with concentration-dependent manner of ODZ10117 (B). (C) Comparison of the effects of ODZ10117 (40 μM) with the known STAT3 inhibitors S3I-201 (100 μM), STA-21 (100 μM), nifuroxazide (NIF, 100 μM), and AG-490 (150 μM) on tyrosine phosphorylation of STAT3. D and E. Breast cancer cells were incubated for 24 h with ODZ10117 (40 μM), S3I-201 (100 μM), STA-21 (100 μM), nifuroxazide (NIF, 100 μM), or AG-490 (150 μM), and then stimulated with IL-6 (20 ng/mL) for 10 min. Immunoblotting was performed using anti-pY-STAT3 or STAT3.

**Supplementary Figure S10.** ODZ10117 does not affect other STAT family members and upstream regulators of STAT3. Human Hodgkin's lymphoma HLDM-2 and L540 cells were incubated for 16 h with ODZ10117 (ODZ, 40 μM) or the known STAT3 inhibitors S3I-201 (100 μM), STA-21 (100 μM), nifuroxazide (NIF, 100 μM), napabucasin (NAPA, 4 μM), or AG-490 (150 μM), and then performed immunoblot analysis.

**Supplementary Figure S11.** ODZ10117 does not affect body weight in the orthotopic metastasis model. Body weight was measured in the tumor-bearing mice, which were generated by injecting MDA-MB-231 (A) or 4T1-Luc cells (B) into the right fourth mammary fat pad of female BALB/c mice.
